# The protozoan parasite *Toxoplasma gondii* encodes a gamut of phosphodiesterases during its lytic cycle in human cells

**DOI:** 10.1016/j.csbj.2020.11.024

**Published:** 2020-11-21

**Authors:** Kim Chi Vo, Özlem Günay-Esiyok, Nicolas Liem, Nishith Gupta

**Affiliations:** a Department of Molecular Parasitology, Institute of Biology, Faculty of Life Sciences, Humboldt University, Berlin, Germany; b Experimental Biophysics, Institute of Biology, Faculty of Life Sciences, Humboldt University, Berlin, Germany; c Department of Biological Sciences, Birla Institute of Technology and Science Pilani (BITS-P), Hyderabad, India

**Keywords:** 3′IT, 3′-insertional tagging, AC, adenylate cyclase, COS, crossover sequence, CRISPR, clustered regularly interspaced short palindromic repeats, EES, entero-epithelial stages, FPKM, fragments per kilobase of exon model per million, GC, guanylate cyclase, GMQE, Global Model Quality Estimation, HFF, human foreskin fibroblast, HXGPRT, hypoxanthine-xanthine-guanine phosphoribosyltransferase, IMC, inner membrane complex, MAEBL, merozoite adhesive erythrocytic binding ligand, MOI, multiplicity of infection, OCRE, octamer repeat, PDE, phosphodiesterase, PKA, protein kinase A, PKG, protein kinase G, PM, plasma membrane, QMEAN, Quality Model Energy Analysis, smHA, spaghetti monster-HA, *sg*RNA, single guide RNA, Apicomplexa, cAMP and cGMP signaling, Lytic cycle, Tachyzoite, Bradyzoite

## Abstract

Cyclic nucleotide signaling is pivotal to the asexual reproduction of *Toxoplasma gondii*, however little do we know about the phosphodiesterase enzymes in this widespread obligate intracellular parasite. Here, we identified 18 phosphodiesterases (*Tg*PDE1-18) in the parasite genome, most of which form apicomplexan-specific clades and lack archetypal regulatory motifs often found in mammalian PDEs. Genomic epitope-tagging in the tachyzoite stage showed the expression of 11 phosphodiesterases with diverse subcellular distributions. Notably, *Tg*PDE8 and *Tg*PDE9 are located in the apical plasma membrane to regulate cAMP and cGMP signaling, as suggested by their dual-substrate catalysis and structure modeling. *Tg*PDE9 expression can be ablated with no apparent loss of growth fitness in tachyzoites. Likewise, the redundancy in protein expression, subcellular localization and predicted substrate specificity of several other PDEs indicate significant plasticity and spatial control of cyclic nucleotide signaling during the lytic cycle. Our findings shall enable a rational dissection of signaling in tachyzoites by combinatorial mutagenesis. Moreover, the phylogenetic divergence of selected *Toxoplasma* PDEs from human counterparts can be exploited to develop parasite-specific inhibitors and therapeutics.

## Highlights

• *Toxoplasma* genome harbors at least 18 phosphodiesterases encoded by distinct genes.
• Most parasite PDEs lack regulatory modules and are quite divergent from their human orthologs.
• Acutely-infectious tachyzoite stage of *T. gondii* expresses 11 PDEs with varied localizations.
• PDE8 and PDE9 are closely-related dual-substrate specific proteins residing in the apical pole.
• Homology modeling of PDE8 and PDE9 reveals a conserved 3D topology and substrate pocket.
• PDE9 is dispensable in tachyzoites, signifying a functional redundancy with PDE8.

## Graphical abstract

**Figure:**
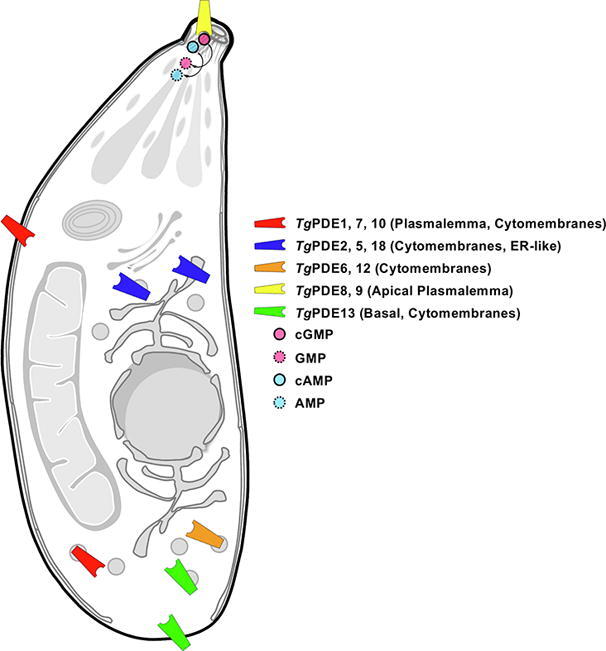

## 1 Introduction

Cyclic nucleotides (cAMP and cGMP) are second messengers which regulate various functions *via* respective signal transduction pathways. The fluctuation in their subcellular concentration causes a cascade of molecular events, eventually leading to a cellular response. Hence, intracellular levels of cyclic nucleotides are strictly counterbalanced. cAMP and cGMP are synthesized by adenylate cyclase (AC) and guanylate cyclase (GC) respectively; conversely, they are degraded into AMP or GMP by phosphodiesterase (PDE) enzymes [1]. The cyclic nucleotide signaling is mostly conveyed by the protein kinases dependent on cAMP (PKA) or cGMP (PKG), which phosphorylate a repertoire of effector proteins. In the protozoan phylum Apicomplexa – a group of >6000 obligate intracellular parasites – the aforesaid signaling proteins are required for the pathogenesis, persistence and inter-host transmission of several members [2], [3], [4]. For instance, in *Plasmodium* and *Toxoplasma* species, the two most prominent members of the phylum, cyclic nucleotides govern many events during their lifecycle; such as, gliding motility, host-cell invasion, intracellular proliferation, stage differentiation, sexual development and lytic egress from host cells, as described below. Consequently, cAMP and cGMP cascade proteins impart excellent drug targets against these clinically-relevant parasitic protists.

In *Plasmodium*, two adenylate cyclases (ACα and ACβ) have been reported [5]; ACα is required for the apical exocytosis in sporozoites and hepatocyte infection [6], whereas ACβ is essential for the erythrocyte invasion, but dispensable for the subsequent developmental stages [7], [8], [9]. Besides, *Pf*PKA was shown to control the invasion of both hepatocytes and erythrocytes [6], [7], [9], [10], [11] but not the cell cycle [9], [11]. Four potential adenylate cyclases (ACα1, ACα2, ACα3 and ACβ) have been reported in *T. gondii*; none of them are essential in the tachyzoite stage [12], however ACβ was suggested to be involved in regulating the host-cell invasion by tachyzoites [13]. cAMP signaling also underlies the acute (tachyzoite) to chronic (bradyzoite) stage switching [14], [15], [16]. Albeit the roles of ACs have not been defined yet, *Tg*PKAc3 (one of the three PKA catalytic subunits in *T. gondii*) was shown to govern the process of stage differentiation [17]. Moreover, *Tg*PKAc1 has been reported to control the premature egress of tachyzoites [13], [18]. Similar to cAMP, cGMP in *Plasmodium* is generated by two guanylate cyclases (GCα and GCβ), each linked with a P4-ATPase domain [5]. *Plasmodium* GCα controls the blood-stage growth [19], [20], [21], and GCβ is critical for the gliding motility and mosquito-midgut invasion by ookinetes [20], [22], [23]. By contrast, there is only one ortholog in *T. gondii* termed ATPase_p_-GC or GC [4], which is essential for the motility-driven invasion and egress in tachyzoites [12], [24], [25], [26]. Both parasites have a single PKG gene to mediate cGMP signaling [27], [28], [29], [30]. *Pf*PKG is essential for many crucial events during the development of *P. falciparum* [2], [31], [32], [33]. Tachyzoites of *T. gondii* on the other hand express *Tg*PKG^I^ and *Tg*PKG^II^ isoforms [28], [34]; however, only knockdown of the former variant phenocopies the *Tg*ATPase_p_-GC mutant [25], [34].

The counter-regulation of cyclic nucleotide signaling in *Plasmodium* is facilitated by four PDEs (α, β, γ, δ). PDEβ is able to hydrolyze both cAMP and cGMP, whereas others are reported as being specific to cGMP [2], [20], [21], [35], [36], [37]. PDEα has two alternatively-spliced isoforms encoded by a single gene, both of which are shown to be nonessential for the erythrocytic growth [36]. PDEβ controls the activation of PKA during invasion of erythrocytes by *Plasmodium* merozoites and quells its activity during early intraerythrocytic growth [35]. PDEγ equilibrates cGMP levels during sporozoite development in mosquito and thereby ensure its transmission to the mammalian host [38]. Finally, PDEδ is dispensable for the erythrocytic cycle but needed by the sexual stages [20], [21]. Unlike *Plasmodium*, the counterbalancing of cAMP and cGMP levels in *T. gondii* is largely unknown. Previous work has indicated the presence of 18 phosphodiesterases [39]. However, none of them have been studied except for *Tg*PDE1 and *Tg*PDE2 which were implied to be essential for the lytic cycle of tachyzoites based on initial attempts to knockout their genes [13]. The occurrence of several yet-enigmatic PDEs coupled with our interest in elucidating the regulation of signaling in tachyzoites of *T. gondii* prompted us to undertake this study. We demonstrate many previously-unknown findings, filling a major gap in our understanding of the parasite biology.

## 2 Results

### 2.1 *Toxoplasma* genome encodes a large repertoire of phosphodiesterases

**Fig. 1:**
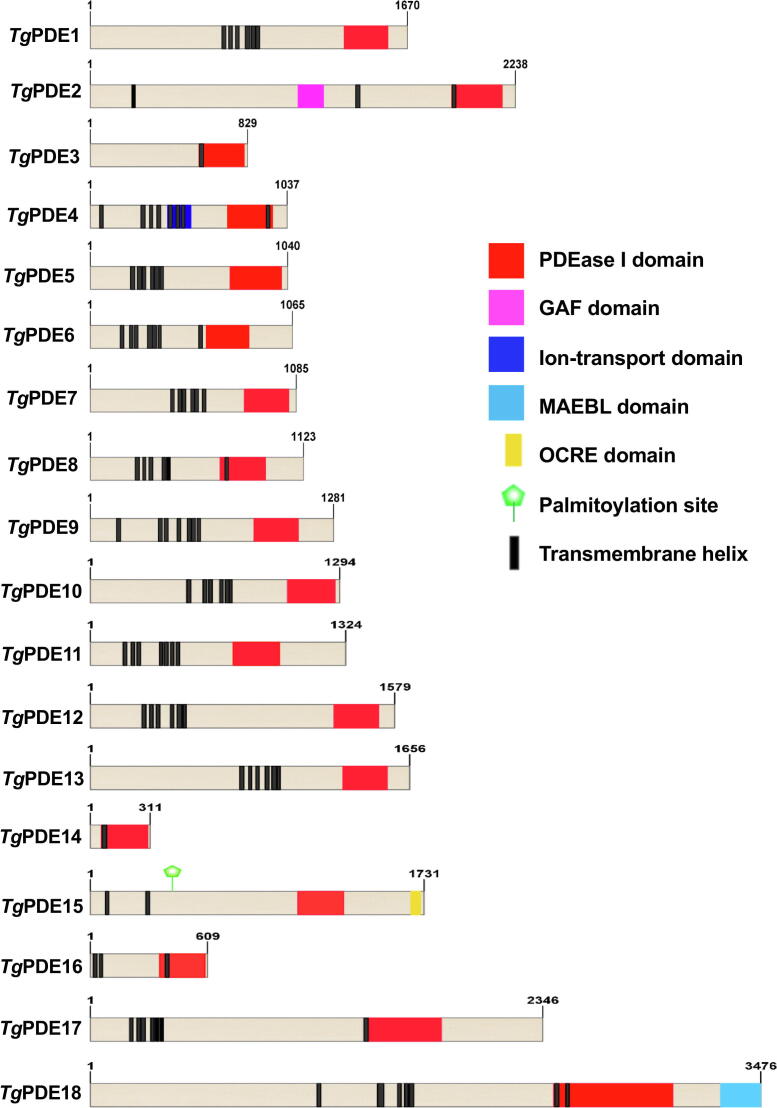
*Toxoplasma gondii harbors 18 cyclic nucleotide phosphodiesterases.* Shown are the primary structure of parasite PDEs. The approximate position of PDEase I domain and other modules were predicted by PFAM, SMART and NCBI conserved domain search tools. The number and location of transmembrane helices are consensus of TMPred, TMHMM and Phobius algorithms. Images were generated with proportional scaling in IBS (v1.0.3) software. PDEase I, domain of cyclic nucleotide phosphodiesterase; GAF, a domain present in certain cGMP-specific PDEs, Adenylyl cyclases and FhlA; OCRE, Octamer repeat; MAEBL, merozoite adhesive erythrocytic binding ligand.

Our genome search identified 18 cyclic nucleotide-specific PDEs in the parasite database (ToxoDB [40]), as also indicated earlier [39]. We named them in a sequential order from *Tg*PDE1-18 including the formerly-reported *Tg*PDE1 and *Tg*PDE2 [13] (Table 1). The pertinent PDE genes are located on different chromosomes throughout the genome. *Tg*PDE14 has the shortest coding region (936 bp), while *Tg*PDE18 comprises the largest open reading frame (10431 bp). The encoded PDE proteins range from 311 to 3476 amino acids, corresponding to a molecular weight of 35-kDa (*Tg*PDE14) and 381-kDa (*Tg*PDE18), respectively. All parasite PDEs harbor a PDEase domain and belong to the class I phosphodiesterase (PDEase I) (Fig. 1) – a shared feature of metazoan enzymes [41]. The predicted PDEase I domain in *Toxoplasma* PDEs consists of 230–280 amino acids except for *Tg*PDE14 (173 aa), *Tg*PDE17 (377 aa) and *Tg*PDE18 (616 aa). Most of the 16 signature residues reported as conserved in all human enzyme families and required for the PDE catalysis were also found in *Tg*PDE1-18 proteins barring *Tg*PDE4 and *Tg*PDE14, which show many substitutions and a large deletion in the catalytic region, respectively (Fig. S1). Each parasite PDE was predicted to harbor at least one transmembrane helix (TM), and therefore all of them appear to be membrane-associated, resonating with *Plasmodium* PDEs and *Hs*PDE3, but contrasting other human orthologs [2], [41], [42], [35], [36], [37].

**Table 1:** Summary of 18 phosphodiesterases present in *Toxoplasma gondii.*

Gene Name (ToxoDB-ID)	Substrate (Predicted)	Protein Size (≈kDa)*	Growth Score (Tachyzoite)**	Transcript (FPKM)***	Expression in Tachyzoites (Immunostaining)
*Tg*PDE1(TGGT1_202540)	Dual	182	−2.43	27.90	PM and Cytomembranes
*Tg*PDE2(TGGT1_293000)	--	244	−2.53	10.84	Cytomembranes/ER-like
*Tg*PDE3(TGGT1_233065)	cGMP	92	−0.71	0.13	Not expressed
*Tg*PDE4(TGGT1_229405)	cAMP	118	−0.35	0.90	Not expressed
*Tg*PDE5(TGGT1_220420)	--	116	1.23	1.81	Cytomembranes/ER-like
*Tg*PDE6(TGGT1_266920)	Dual (?)	120	1.63	159.84	Cytomembranes
*Tg*PDE7(TGGT1_280410)	Dual	122	0.70	14.39	PM and Cytomembranes
*Tg*PDE8(TGGT1_318675)	Dual****	127	0.19	1.33	Apical PM and Cytomembranes
*Tg*PDE9(TGGT1_241880)	Dual****	142	0.52	38.25	Apical PM
*Tg*PDE10(TGGT1_272650)	cAMP	141	−0.37	21.79	PM and Cytomembranes
*Tg*PDE11(TGGT1_224840)	cAMP	147	0.40	12.22	Not expressed
*Tg*PDE12(TGGT1_310520)	Dual	174	−0.06	3.05	Cytomembranes (granular)
*Tg*PDE13(TGGT1_257080)	cGMP	180	0.55	21.98	Basal and Cytomembranes
*Tg*PDE14(TGGT1_228500)	cGMP	35	−0.33	0.29	Not expressed
*Tg*PDE15(TGGT1_233040)	–	195	1.22	0.30	Not expressed
*Tg*PDE16(TGGT1_258508)	cAMP	68	−1.19	0.92	Not expressed
*Tg*PDE17(TGGT1_257945)	–	254	−1.81	10.03	Not expressed
*Tg*PDE18(TGGT1_226755)	cGMP	381	−1.15	2.37	Cytomembranes/ER-like

*Theoretical molecular weight of the open reading frames without any post-translational modification and epitope-tagging. ** CRISPR/Cas9-assisted genome-wide knockout screening [59]. A negative score means an impaired growth.

***FPKM (fragments per kilobase of exon model per million) in tachyzoites [53].

****Confirmed by PDE enzyme assay (*see text*).

A distinguished feature of most parasite PDEs is the general lack of archetypal regulatory domains (Fig. 1). Such domains (*e.g*. calmodulin-binding, GAF, PAS or REC) are known to facilitate the subcellular trafficking, substrate affinity and catalytic activity of metazoan PDEs [42]. We identified an ancillary module in only 4 out of 18 parasite proteins (Figs. 1, S2). *Tg*PDE2 possesses a GAF-like domain (expect value: 3.43e^-04^, *high probablity*), which usually binds cGMP and thereby controls the PDE catalysis [43]. Equally, *T*gPDE4 harbors a channel-like ion-transport domain (expect value: 4.53e^−09^, *high probablity*) embedded in three TM helices (Fig. 1, S2). *Tg*PDE15 has an octamer repeat (OCRE, 42 aa, expect value: 1.04e^-03^, *high probablity*) at the C-terminal, and *Tg*PDE18 comprises a domain analogous to the merozoite adhesive erythrocytic binding ligand (MAEBL, expect value: 3.88e^−05^, *high probablity*). Furthermore, a palmitoylation site was found in *Tg*PDE15 based on the reported palmitome of *T. gondii* tachyzoites [44]. Not least, PDEase I and apposite secondary domains in *Tg*PDE2, *Tg*PDE15 and *Tg*PDE18 are topologically oriented for a mutual functioning of their *catalytic* and *regulatory* regions (Fig. S2).

### 2.2 Most apicomplexan PDEs are not related to the metazoan orthologs

**Fig. 2:**
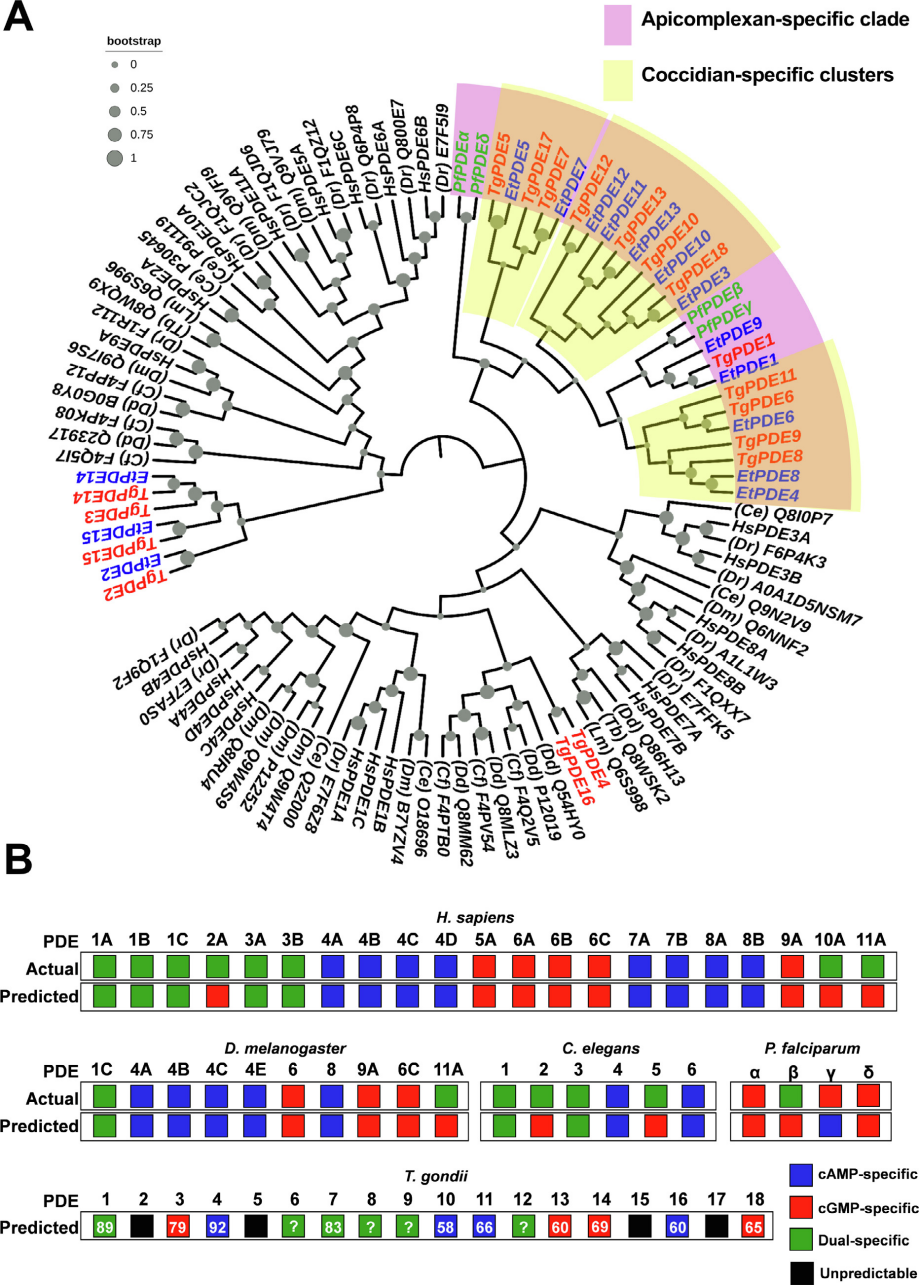
Most *Toxoplasma* PDEs group with other apicomplexan phosphodiesterases and are predicted to degrade cAMP and/or cGMP. (A) A parsimonious phylogenetic tree of the PDEase domains from *Tg*PDE1-18 with their orthologs from *Homo sapiens* (*Hs*), *Drosophila melanogaster* (*Dm*), *Danio rerio* (*Dr*), *Caenorhabditis elegans (Ce*), *Dictyostelium discoideum* (*Dd*), *Cavenderia fasciculata* (*Cf*), *Leishmania major* (*Lm*), *Trypanosoma brucei* (*Tb*), *Eimeria tenella* (*Et*) and *Plasmodium falciparum* (*Pf*). The cladistic analysis was performed using the Maximum Likelihood method (1000 bootstraps, gray spheres). Accession numbers (UniprotKB) along with the organism abbreviations are indicated in brackets except for human and apicomplexan PDEs (see Table S1; *Tg*, red; *Et*, blue; *Pf,* green). (B) Substrate specificity of PDE proteins, as predicted by their individual clustering with cAMP-, cGMP- and dual-specific consensus sequences of human PDEs (Fig. S1), or *Pf*PDEs. The bootstrap score of individual clades (if acquired) are shown only for *Toxoplasma* PDEs to minimize *numerical cluttering*. The question-marked boxes indicate a bootstrap ≤ 50 (green or red), and the black boxes refer to outgroup clading. (For interpretation of the references to color in this figure legend, the reader is referred to the web version of this article.)

Similar to *T. gondii*, our search of *Eimeria tenella*, a related coccidian parasite, identified 15 putative PDEs encoded by its genome (Table S1), which are termed here as *Et*PDE1-15 based on their pairwise similarities with *Tg*PDE1-18 proteins. To understand their evolutionary relationship, we performed a phylogenetic clading with other phosphodiesterases from protozoan, invertebrate, as well as vertebrate organisms (Fig. 2A). A parsimonious cladogram of the catalytic domains resulted in 1 large and 2 small branches of apicomplexan PDEs. One such clade included a majority of apicomplexan PDEs (purple color, Fig. 2A), illustrating their significant divergence from metazoan orthologs. It consists of 4 explicit clusters discounting an outgroup of *Pf*PDEα and *Pf*PDEδ. The first cluster grouped *Pf*PDEβ and *Pf*PDEγ and some *Toxoplasma* and *Eimeria* orthologs. The remaining three clusters on the other hand contained only coccidian PDEs (yellow colored). Equally, some other PDEs (*Tg*PDE2 and 3, *Et*PDE2, *Tg*PDE14 and 15, *Et*PDE14 and 15) diverged off the mainstream proteins and generated yet-another coccidian-specific clade. Unexpectedly, *Tg*PDE4 and *Tg*PDE16 formed a cluster with the slime mold PDEs. Phylogenetic analysis of the full-length PDE sequences resulted in a broadly similar topology and segregation except for few exceptions (Fig. S3). Here, we observed a large protozoan-specific clade, which comprised most apicomplexan proteins. Yet again, *Tg*PDE2,3 and *Tg*PDE14,15 along with *Tg*PDE17 were separated from the large clade, forming small clusters. Finally, the pairwise sequence analysis of *Tg*PDE1-18 proteins resonate rather well with aforementioned phylogenetic analyses (Fig. S4). Particularly, we noted that *Tg*PDE3 and *Tg*PDE14 are most identical to each other (77%), whereas *Tg*PDE4 and *Tg*PDE14 are the most distantly related PDEs (19%) among all *Toxoplasma* proteins.

### 2.3 Substrate specificity of *Tg*PDE1-18 as predicted by *in silico* analysis

PDE enzymes of different families display significant variation in the composition of key constituent residues, which cumulatively determine the shape, size and polarity of the individual catalytic pocket [45], [46]. The substrate specificity of PDEs is thus governed by coordinated interaction of many residues [45]. Following this notion, the catalytic preference of *Tg*PDE1-18 was predicted by clustering them with the signature consensus sequences of 21 human PDEs (Fig. S1) grouped according to their substrate affinity toward cAMP and/or cGMP [41], [42], [47]. To endorse our method, we first gauged the underlying human PDEs. Indeed, the predictions of all but a few dual-specific PDEs (*Hs*PDE2A, *Hs*PDE10A and *Hs*PDE11A) were correctly matched to their actual substrate specificity (Fig. 2B). Moreover, of the 16 well-described invertebrate PDEs, 12 were assigned their accurate substrate, as reported experimentally [48], [49], [50], [51]. Yet again, only dual-specific *Dm*PDE11, *Ce*PDE2 and *Ce*PDE5 were falsely judged to be *cGMP-specific* (Fig. 2B). We also noted that misprediction of these dual-specific PDEs could in fact be ascribed to their higher affinity towards cGMP than cAMP [48], [50]. Unlike metazoan PDEs (80% accuracy), only 2 of the 4 *Plasmodium* PDEs were classified correctly with their *bona fide* substrates [2], [20], [21], [35], [36], [37]. We therefore adapted a fusion approach based on both human and *Plasmodium* PDEs to evaluate the catalytic activity of *Tg*PDE1-18.

We were able to predict the substrates of most *Toxoplasma* PDEs (Fig. 2B). *Tg*PDE3, *Tg*PDE13, *Tg*PDE14 and *Tg*PDE18 are expected to hydrolyze cGMP; whereas *Tg*PDE4, *Tg*PDE10, *Tg*PDE11, and *Tg*PDE16 were grouped as *cAMP-specific*. *Tg*PDE1, *Tg*PDE6-9 and *Tg*PDE12 were predicted as being *dual-specific*. Given the misprediction of dual-specific human PDEs, we primarily relied on *P. falciparum* PDEs to assess the *dual-specific Toxoplasma* proteins. It is worth noting that *Tg*PDEs projected as *cAMP-specific* based on their clading with human sequences formed outgroups with *Pf*PDEs, likely due to lack of a *cAMP-specific* enzyme in the latter parasite. *Tg*PDE18 as an exception exhibited inverse clading patterns with cGMP-specific *Pf*PDEα (65% bootstrap) and cAMP-consensus human sequence (52% bootstrap), of which we considered the former analysis. *Tg*PDE8 and *Tg*PDE9 by contrast were classified as *dual-specific* by both approaches but not supported by a bootstrap value (*question-marked* in Fig. 2B). On the other hand, *Tg*PDE2, *Tg*PDE5, *Tg*PDE15 and *Tg*PDE17 did not explicitly clade with human consensus or *Pf*PDE sequences; hence only 14 *Toxoplasma* PDEs could be eventually assigned a substrate with reasonable confidence.

### 2.4 At least 11 PDEs are expressed during the lytic cycle of *T. gondii*

**Fig. 3:**
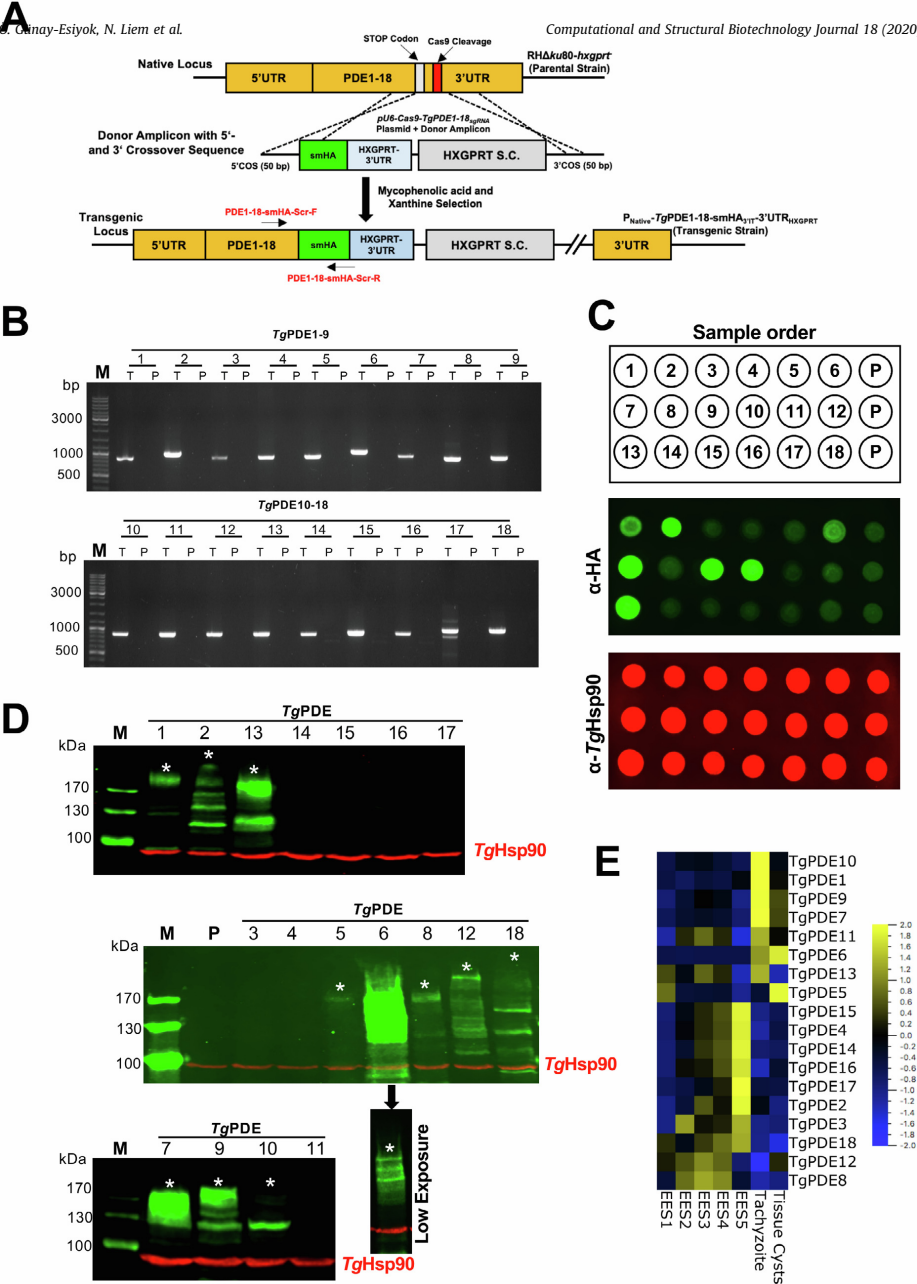
*TgPDE1-18 are expressed in a broadly stage-specific manner during the lifecycle of T. gondii*. (A) Scheme illustrating the 3′-insertional tagging (3′IT) of *Tg*PDE1-18 genes with *spaghetti monster* (smHA) epitope. For each PDE, a plasmid encoding Cas9 and gene-specific guide RNA (*pU6-Cas9-TgPDE1-18_sgRNA_*) was constructed and transfected along with the corresponding donor amplicon into the RHΔ*ku80/hxgprt^–^* (parental) strain. Transgenic parasites were selected for HXGPRT expression (selection cassette, S.C.) (B) Genomic screening of the clonal strains expressing individual *Tg*PDE1-18-smHA_3′IT_ proteins. Screening primers, specified as red-labeled arrows in *panel A*, were used to test gDNA from the transgenic (“T”) and parental (“P”, *negative control*) strains. (C, D) Immuno-dot and western blots of tachyzoites encoding smHA-tagged PDEs. The protein extracts were either directly loaded onto blotting membrane (C), or first resolved by 8% SDS-PAGE and then blotted (D). In both cases, blots were subjected to immunostaining for the HA-tag along with *Tg*Hsp90 (loading control). Note that the appearance of 4 extra PDEs (*Tg*PDE5, 8, 12 and 18) in the western blot is due to 4× higher sample loading than dot blot. Visualization of the bands of expected theoretical molecular weight for *Tg*PDE5, 8, 10, 12 and 18 requires a very high contrast and exposure, which oversaturates other PDEs including *Tg*PDE6, 7 and 9. Hence, western blot does not allow a fair comparison of relative expression levels, which can be better assessed by dot blot. Sample loading in dot blot follows the PDEs, as numbered, and parental strain (“P”) as a negative control. (E) Heat map showing transcript expression (FPKM values) of *Tg*PDE1-18 in tachyzoites, tissue cysts and entero-epithelial stages (EES1-EES5 [53]). EES1 = very early, EES2 = early, EES3 = mixed, EES4 = late, EES5 = very late. (For interpretation of the references to color in this figure legend, the reader is referred to the web version of this article.)

We examined the expression of *Tg*PDE1-18 in tachyzoites following CRISPR/Cas9-assisted 3′-genomic tagging with a *spaghetti monster*-HA (smHA)-epitope (Fig. 3A), which enables a detection of even poorly-expressed proteins [52]. PCR screening with recombination-specific primers and subsequent sequencing of amplicons confirmed successful smHA-tagging of 18 genes in clonal transgenic strains (Fig. 3B). Immuno-dot blot of epitope-tagged tachyzoites revealed a strong expression of at least 7 PDEs (*Tg*PDE1, 2, 6, 7, 9, 10, 13) (Fig. 3C). The western blot disclosed the expression of 4 additional proteins (*Tg*PDE5, 8, 12, 18) (Fig. 3D). All of the 11 PDEs displayed a band of their theoretical protein size (see *asterisk-marks* in Fig. 3D). The predicted bands of *Tg*PDE5, 8, 10, 12 and *Tg*PDE18 were visible only after much high exposure and contrast. In some cases (*Tg*PDE2, 6, 9, 12, 13, 18), extra protein bands of lower size were also seen that can be attributed to degradation, processing, alternative translation and/or splicing variants. Expression of *Tg*PDE3, 4, 11, 14–17 were not evident by any immunostaining method. Our protein expression results correlated with the transcription of PDEs in tachyzoites (Fig. 3E, [53]). As such, transcripts of well-stained PDEs are overexpressed, while those not detectable or weakly stained by immunoblot are underexpressed during acute stage (Fig. 3D-E, Table 1). In contrast, proteins absent in tachyzoites displayed much higher transcript level during entero-epithelial stages (EES1-5) of the parasite in its felid host. Furthermore, transcripts of *Tg*PDE5 and *Tg*PDE6 were markedly overexpressed during chronic infection (Fig. 3E).

### 2.5 Phosphodiesterases expressed in tachyzoites show assorted localizations

**Fig. 4:**
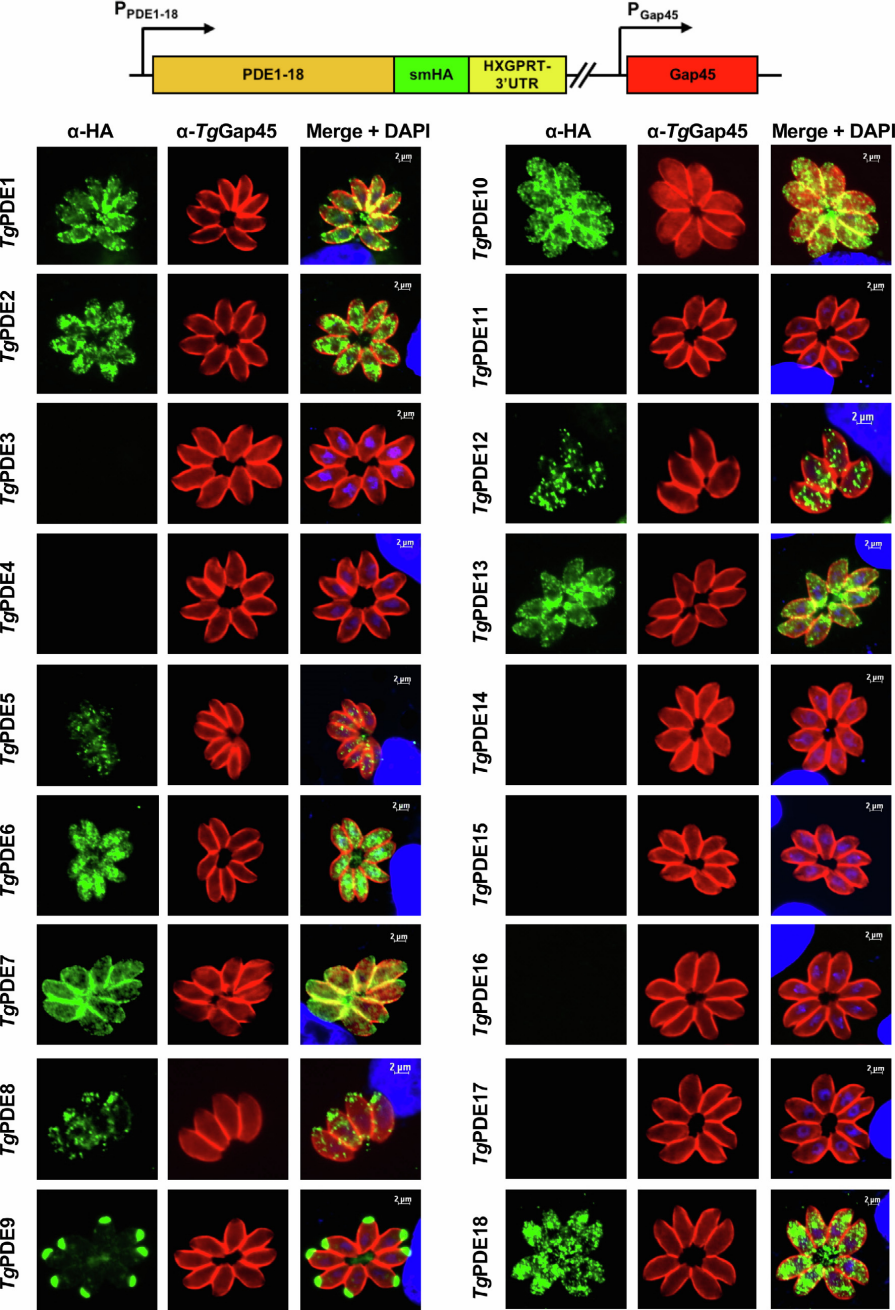
*Phosphodiesterases expressed in tachyzoites show assorted spatial distribution*. Intracellular parasite strains encoding individual smHA-tagged *Tg*PDE1-18 proteins (*Tg*PDE1-18-smHA_3′IT_) were allowed to infect confluent HFF cells (24–36 h post-infection), and then immunostained using α-HA and α-*Tg*Gap45 antibodies. The host-cell and parasite nuclei were visualized by DAPI (scale, 2 μm). Images were acquired using clonal transgenic tachyzoites (Fig. 3). Panels with no detected smHA staining denote a lack of protein expression. In case of *Tg*PDE5, 8 and 18, only a minor fraction of vacuoles was fluorescent that is probably due to dependence on the cell cycle and/or low transcript expression (Fig. S5A and Table 1).

To gain added insight into cyclic nucleotide signaling, we localized all PDEs by immunofluorescence staining of the transgenic strains expressing smHA-tagged proteins (Fig. 4). As anticipated, only those proteins visible in immunoblot showed a fluorescence signal. *Tg*PDE3, 4, 11 and *Tg*PDE14-17 were not detectable, confirming a lack of their expression in the tachyzoite stage. Of remaining 11 proteins, *Tg*PDE2, 5, 6, 12, 13 and *Tg*PDE18 demonstrated punctate cytosolic (cytomembranes) distribution in tachyzoites, whereas *Tg*PDE1, *Tg*PDE7 and *Tg*PDE10 were present at the periphery alongside granulated expression throughout the parasite body (Fig. 4). *Tg*PDE13 was evidently more confined to the basal end. *Tg*PDE8 and *Tg*PDE9 displayed a conspicuous apical staining. The former also disclosed a punctuate cytosolic expression, and it was not expressed in all parasites of a clonal population (Fig. S5A). Evenly, the clonal transgenic strains harboring *Tg*PDE5 and *Tg*PDE18 exhibited cytomembrane signal, and were not uniformly immunostained (Figs. 4, S5A). Importantly, the FPKM values of *Tg*PDE5, *Tg*PDE8 and *Tg*PDE18 transcripts range ≈1-2 in tachyzoites (Table1), which seems to be the sensitivity threshold of high-affinity smHA tagging because any other PDEs < 1 FPKM were not detectable at the protein level (Fig. 3, Fig. 4). *Tg*PDE11 and *Tg*PDE17 with FPKM > 10, yet not detectable by immunostaining, were the only exceptions to this observation, which might be subjected to post-transcriptional regulation of protein expression in tachyzoites.

### 2.6 *Tg*PDE8 and *Tg*PDE9 localize in the apical plasmalemma of tachyzoites

**Fig. 5:**
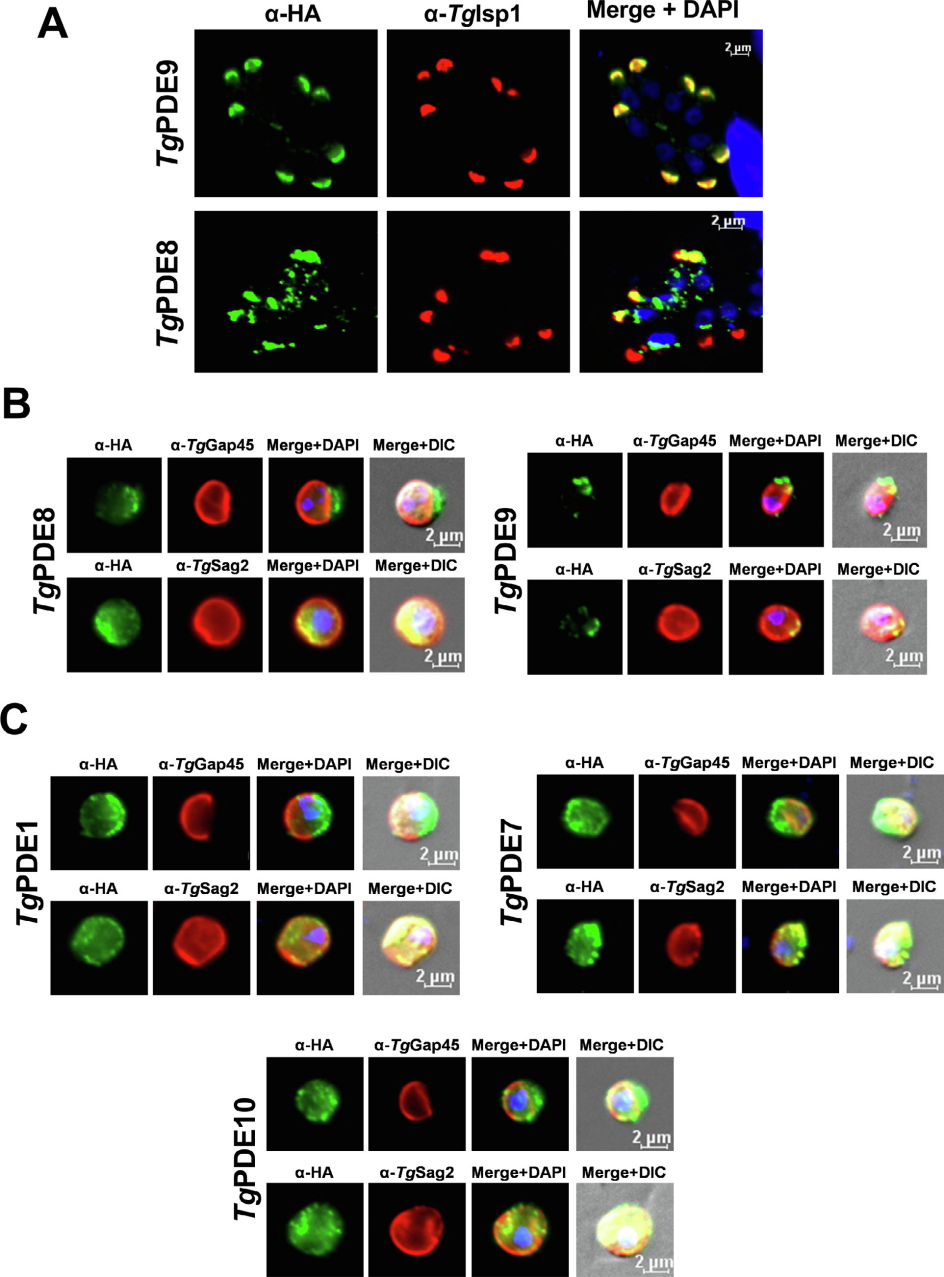
*TgPDE8 and TgPDE9 reside in the apical plasmalemma, whereas TgPDE1, TgPDE7 and TgPDE10 are located* at the periphery *of tachyzoites.* (A) Localization of *Tg*PDE8 and *Tg*PDE9 with an apical marker (*Tg*Isp1). Intracellular parasites expressing *Tg*PDE8-smHA_3′IT_ and *Tg*PDE9-smHA_3′IT_ were stained with α-HA and α-*Tg*Isp1 antibodies. (B, C) Immunostaining of smHA-tagged *Tg*PDE8 and *Tg*PDE9 (B), and of *Tg*PDE1,*Tg*PDE7 and *Tg*PDE10 (C) in tachyzoites treated with α-toxin. Inner membrane complex and plasma membrane were stained by α-*Tg*Gap45 or α-*Tg*Sag2 antibodies, respectively, after drug-induced uncoupling of the two entities (see control assays in Fig. S5B). The merged images include DAPI-stained host and parasite nuclei in blue (scale, 2 μm). (For interpretation of the references to color in this figure legend, the reader is referred to the web version of this article.)

A prominent expression of *Tg*PDE8 and *Tg*PDE9 at the apex of tachyzoites prompted us to perform additional immunofluorescence assays to define their precise localization. As shown in Fig. 5A, both PDEs co-localized with inner membrane complex sub-compartment protein 1 (*Tg*Isp1) – a *bona fide* marker of the apical region in tachyzoites [54]. Immunostaining of *Tg*Isp1 completely intersected with *Tg*PDE9 and partially overlapped with *Tg*PDE8 (Fig. 5A) in accordance with their expression in intracellular parasites (Fig. 4). Given multiple TMs in *Tg*PDE8 and *Tg*PDE9 (Fig. 1), we tested their association with the plasma membrane (PM) and/or inner membrane complex (IMC) following α-toxin-induced membrane splitting in tachyzoites (Fig. 5B). As expected, the drug-treated control samples revealed a clear separation of IMC and PM, ascertaining the functionality of our assay (Fig. S5B). Similarly, a co-staining of *Tg*PDE8 and *Tg*PDE9 with *Tg*Sag2 disclosed their presence in the plasma membrane (Fig. 5B). Using the same technique, we investigated the exact membrane distribution of *Tg*PDE1, *Tg*PDE7 and *Tg*PDE10, which appeared peripheral in intracellular parasites (Fig. 4). Indeed, all 3 PDEs co-localized primarily with *Tg*Sag2 after α-toxin-induced membrane splitting, advocating their expression in the plasmalemma (Fig. 5C).

### 2.7 Modeling of *Tg*PDE8 and *Tg*PDE9 suggests a prototypical 3D structure

**Fig. 6:**
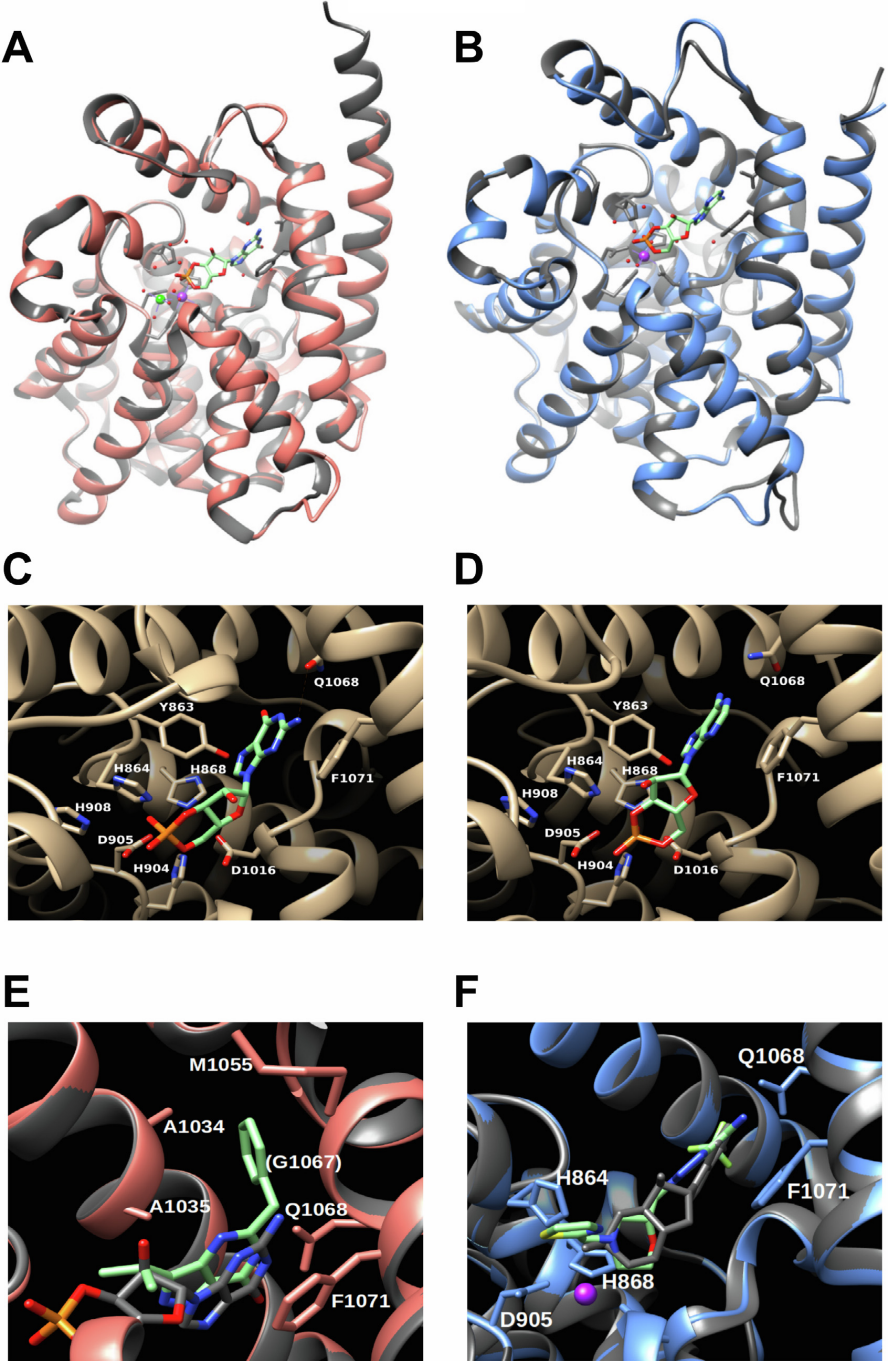
*TgPDE9 exhibits a typical tertiary structure with a defined substrate pocket*. (A, B) Homology models of *Tg*PDE9 overlaid with the crystal structure of *Hs*PDE9A (PDB, 3dyn) (A) and of *Hs*PDE4D (PDB, 2pw3) (B). Human PDEs are depicted in gray background and corresponding models of the *Tg*PDE9 catalytic domain are illustrated in salmon and blue, respectively. Zn^2+^ (purple) and another metal ion (*e.g.*, Mg^2+^, green) bound to the catalytic center are also shown as spheres. (C, D) Inset view of the substrate-binding pocket of *Tg*PDE9 with cGMP (C) and cAMP (D), as deduced by modeling based on *Hs*PDE9A and *Hs*PDE4D, respectively. Only key residues located in the catalytic site are indicated. (E) BIPPO-docked *Tg*PDE9 model (salmon) superimposed with cGMP-bound *Hs*PDE9A (gray). (F) Docking of PF-04957325 into *Tg*PDE9 model (blue) overlaid with cAMP-bound *Hs*PDE4D (gray). (For interpretation of the references to color in this figure legend, the reader is referred to the web version of this article.)

Subcellular expression of *Tg*PDE8 and *Tg*PDE9 suggested their involvement in the apical signaling, which is initiated by apically-located P4-ATPase-conjugated guanylate cyclase [12], [24], [25], [26]. Hence, we first modeled *Tg*PDE8 and *Tg*PDE9 based on the crystal structures of well-characterized cGMP-specific *Hs*PDE9A and cAMP-specific *Hs*PDE4D to discern their enzymatic features (Figs. 6, S6). The tertiary topology of both parasite PDEs was found similar to *Hs*PDE9A (Fig. 6A, S6A) and *Hs*PDE4D (Fig. 6B, S6B). As known in other class I phosphodiesterases, the catalytic domain of *Tg*PDE8 and *Tg*PDE9 comprises 16 α-helices with a deep pocket for the substrate binding (Fig. S7). The region between helix 6 and 15 contains residues dictating the catalytic activity of PDEs [41]. Indeed, we observed that most of the metal-binding and substrate-recognition residues are conserved in *Tg*PDE8 and *Tg*PDE9. Moreover, the H-(X)_3_-H-(X)_25-35_-(D/E) signature of class I enzymes [55] is also present in both PDEs (Fig. S7).

Homology models of *Tg*PDE8 and *Tg*PDE9 allowed the docking of cGMP as well as cAMP into their substrate-binding pockets (Fig. S6C-D, Fig. 6C-D). The phosphate group of cyclic nucleotides faces the putative catalytic center, essentially formed by the histidine and aspartate residues serving metal-binding and proton-donating roles [56]. The catalytic center of *Tg*PDE8 is formed by His685, His689, His725, Asp726, His729 and Asp838 residues (Fig. S6C-D, S7). Besides, the steric constrain and a polar interaction are realized by Tyr684 and its OH-group. In *Tg*PDE9, these residues are identified as Tyr863, His864, His868, His904, Asp905 and His908 (Fig. 6C- D, S7). The classical invariant glutamine – a hallmark of cyclic nucleotide phosphodiesterases [45], [47], [57], [58] – is also present in *Tg*PDE8 (Gln890) (Fig. S6C-D) and in *Tg*PDE9 (Gln1068) (Fig. 6C-D), where it is poised to stabilize the purine moiety in cAMP or cGMP *via* hydrogen-bond networks. Further, the substrate-pocket of *Tg*PDE8 and *Tg*PDE9 can accommodate the purine moiety *via* π-stacking with Phe893 and Phe1071, respectively (Figs. S6C-D, 6C-D, S7). Although not applicable to all PDE families [45], the selection of cAMP and/or cGMP in *Tg*PDE8 and *Tg*PDE9 may hinge upon the orientation of glutamine, as described elsewhere [58]. In brief, the conserved topology, signature residues, substrate specificity and apical location entail a role of *Tg*PDE8 and *Tg*PDE9 in cAMP and cGMP signaling.

### 2.8 Interaction of PDE inhibitors with *Tg*PDE8 and *Tg*PDE9

A range of PDE inhibitors have been developed, of which zaprinast and BIPPO are widely used to artificially induce cGMP signaling in apicomplexan parasites [37], [39]. Using *Hs*PDE9A template, we observed that BIPPO inhibitor-bound *Tg*PDE9 adopted a conformation similar to *Hs*PDE9A (Fig. 6E), as shown by *Howard et al*. [39]. The purine ring of BIPPO mimics the substrate binding and faces parallel to the Phe1071. Furthermore, a hydrophobic pocket is formed by Ala1034, Ala1035 and Met1055 residues, accommodating the additional benzene moiety of BIPPO. Of note, the smaller side chain of Gly1067 precludes any steric hindrance. In contrary, the *Hs*PDE4D-based homology model did not allow an analogous conformation, confirming that docking of inhibitors depends on the previous experimental knowledge of substrate specificity and modeling template. BIPPO could also not be docked on *Tg*PDE8 model with confidence due to bulky Cys889 residue positioned in its catalytic pocket (Fig. S6E), which probably prevents the sandwiching of the benzene ring as depicted in *Tg*PDE9 (Fig. 6E) as well as in *Pf*PDEβ models [39]. The simulated binding of PF-04957325 (inhibitor of cAMP-PDEs) to *Tg*PDE9 model with *Hs*PDE4D template showed that the drug could bind *via* π-stacking with Phe1071 similar to the purine moiety of cAMP, and a polar interaction between its trifluoromethyl group and Gln1068 should augment the binding (Fig. 6F). Its bulky side chain would thereby block the substrate entry into its pocket and to the catalytic center surrounded by His864, His868 and Asp905. Our simulation of *Tg*PDE8 with PF-04957325 yielded analogous results (Fig. S6F).

### 2.9 *Tg*PDE8 and *Tg*PDE9 are dual-specificity PDEs, and *Tg*PDE9 is not required for the lytic cycle

**Fig. 7:**
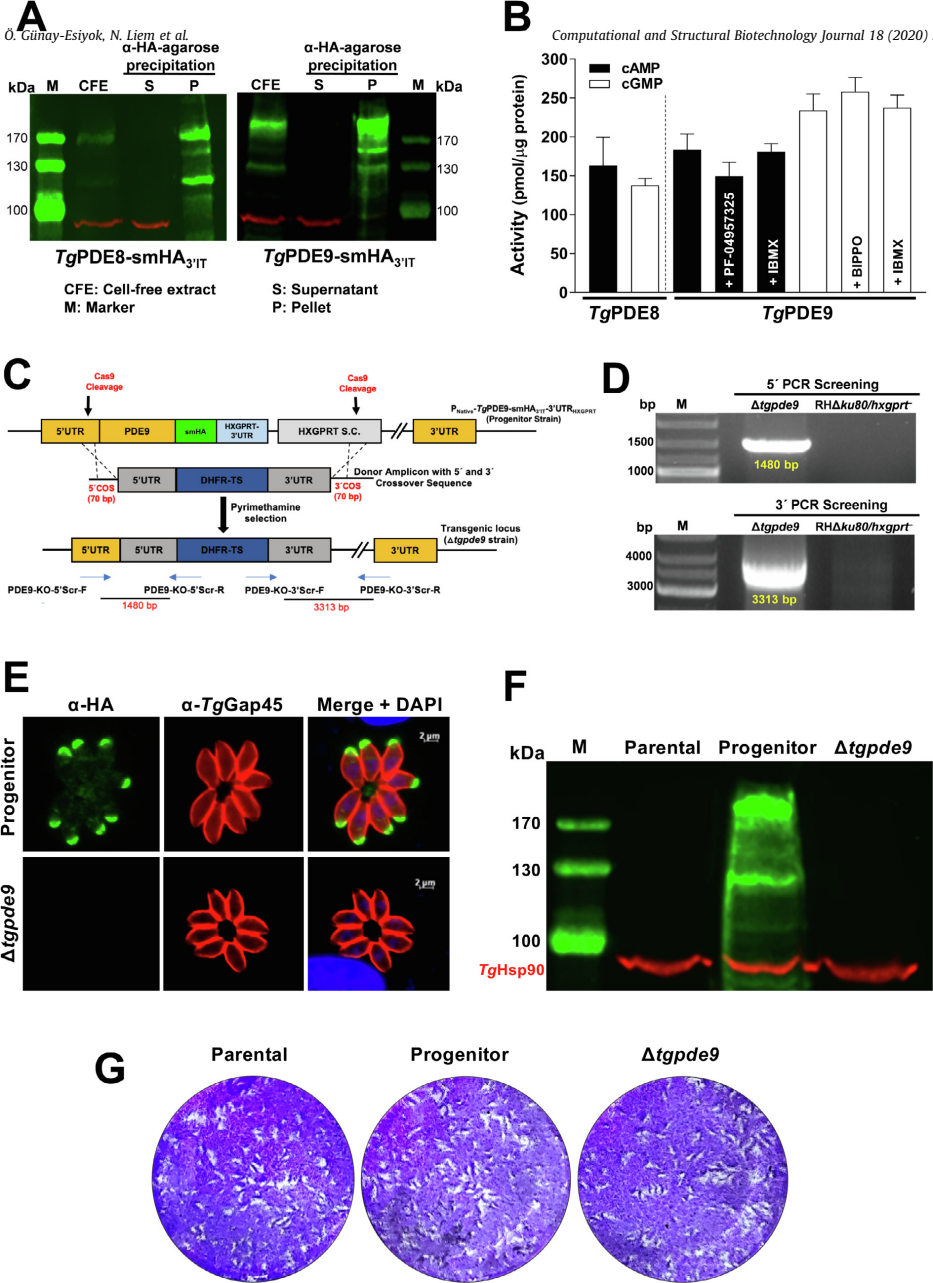
*TgPDE8 and TgPDE9 can hydrolyze both cAMP as well as cGMP; and tachyzoites can survive a knockout of the latter enzyme.* (A) Immunoprecipitation of the native *Tg*PDE9-smHA_3′IT_ protein by means of α-HA-agarose beads. Fresh syringe-released tachyzoites (1 × 10^9^ for *Tg*PDE8; 2 × 10^8^ for *Tg*PDE9) were used to prepare indicated samples. Note that a much higher amount of tachyzoites was required for *Tg*PDE8 due to its very low and heterogeneous expression (see Fig. 3C-D, S5A). *Tg*Hsp90 (red), a cytosolic protein, was used as a control. (B) Colorimetric phosphodiesterase activity assay to test the functionality and substrate specificity of *Tg*PDE8 and *Tg*PDE9. The enzyme assay was set up using 200 μM of substrate (cAMP or cGMP) and 6-22 μg protein. The common PDE inhibitors (BIPPO, 100 μM; IBMX, 50 μM; PF-04957325, 10 μM) were included, as indicated. The control reaction did not contain any enzyme but comprised the corresponding substrates. The substrate-free (enzyme-only) reaction was subtracted to measure the hydrolytic activity, which was normalized to the BCA-quantified protein amounts. (C) Schematics of *Tg*PDE9 knockout by CRISPR/Cas9-assisted double homologous recombination in smHA-tagged progenitor strain (see Fig. 3A). Transgenic mutant parasites were selected by pyrimethamine. (D) Genomic PCR using crossover-specific primers confirming the events of 5′ and 3′ recombination in the Δ*tgpde9* mutant. (E, F) Immunofluorescence and immunoblot of tachyzoites proving a loss of apical signal in the Δ*tgpde9* strain. *Tg*Gap45 (immunofluorescence) and *Tg*Hsp90 (immunoblot) were visualized as control proteins. (G) Plaque assays demonstrating the growth fitness of specified strains in standard culture medium. The assay was set up by infecting confluent HFF monolayers with 150 parasites of each strain (7 days) (n=3 assays).

Next, we determined the actual substrate specificity of *Tg*PDE8 and *Tg*PDE9 proteins. In this regard, we immunoprecipitated smHA-tagged native enzymes from the parasite extract and subjected them to phosphodiesterase assay (Fig. 7A-B). Both immunoprecipitated proteins revealed expected bands along with minor degradation products in western blot (Fig. 7A). We observed that *Tg*PDE8 and *Tg*PDE9 were catalytically active with both cAMP as well as cGMP. The activity of *Tg*PDE9 for cGMP was almost 2x higher than *Tg*PDE8, whereas the cAMP hydrolysis by two enzymes was fairly comparable (Fig. 7B). A weak and heterogeneous expression of *Tg*PDE8 in tachyzoites prevented us from characterizing it further. However, we did set up additional assays with *Tg*PDE9 aimed at measuring the enzyme inhibition by cAMP-specific PF-04957325, cGMP-specific BIPPO and dual-specific IBMX. We found that *Tg*PDE9 activity was not affected by BIPPO (100 μM) and IBMX (50 μM) treatment, while a modest decline in cAMP hydrolysis was observed upon inclusion of PF-04957325 (10 μM) (Fig. 7B).

Finally, to examine the physiological importance of *Tg*PDE9, we generated a direct knockout mutant of *Tg*PDE9 by CRISPR/Cas9-assisted homologous crossover (Fig. 7C). Genomic screening using 5′- and 3′-recombination-specific primers confirmed a successful gene deletion in the Δ*tgpde9* strain (Fig. 7D), which was validated by sequencing of the PCR products. Immunofluorescence staining confirmed a loss of *Tg*PDE9 expression at the apical pole of the mutant tachyzoites (Fig. 7E). In accord, the protein was not detectable in the immunoblot analysis of the Δ*tgpde9* mutant (Fig. 7F). The plaque assay, which signifies the overall fitness of tachyzoites during serial lytic cycles, disclosed a normal growth of the Δ*tgpde9* mutant when compared to the parental and progenitor strains (Fig. 7G). These and above findings, taken together, suggest a functional redundancy of *Tg*PDE8 and *Tg*PDE9 proteins during the lytic cycle of *T. gondii* tachyzoites.

## 3 Discussion

This study demonstrates that out of 18 class I PDE proteins present in *Toxoplasma gondii* at least 8 PDEs are expressed throughout the lytic cycle of tachyzoites, while the expression of 3 others seems to oscillate through the cell cycle, and the remaining 7 proteins are not detectable. Those translated in tachyzoites display varied spatial distributions including plasmalemma, ER-like and cytomembranes. *Tg*PDE8 and *Tg*PDE9 localize at the apical pole, where they probably offset cAMP and cGMP signaling to control the motility, invasion and egress events in a spatiotemporal manner. Moreover, despite being phylogenetically distinct from human PDEs, the catalytic domains of *Tg*PDE8 and *Tg*PDE9 embrace a surprisingly exemplary tertiary topology. The redundancy observed in expression, localization and predicted substrate specificities of tachyzoite-encoded PDEs suggests a significant plasticity in the regulation of parasite signaling. We also noted an evolutionary divergence of coccidian PDEs including *Eimeria*, which harbors at least 15 PDEs similar to *T. gondii*. Unlike other apicomplexans, such a large gamut of PDEs in coccidian parasites signifies their possibly redundant and specialized functions during the lifecycle.

The RNA sequencing of the major lifecycle stages in *T. gondii* indicates stage-specific expression of many PDEs [53], whereas the CRISPR/Cas9-assisted genome-wide mutagenesis screen suggests several PDEs as nonessential in tachyzoites [59] (Table 1). Our findings revealing the expression of selected PDEs (but not of others) are broadly consistent with these transcriptomic and phenotypic datasets. Excluding *Tg*PDE1, *Tg*PDE2, *Tg*PDE16 and *Tg*PDE18, other PDEs including *Tg*PDE9 show none to modest defect upon ablation in tachyzoites. *Tg*PDE1 and *Tg*PDE2 were projected as essential in a study focused on coordinated regulation of PKA and PKG activity [13]. Based on the predicted *dual-specificity*, transcriptional upregulation and peripheral location, we propose *Tg*PDE1 (and *Tg*PDE7) to calibrate cAMP and cGMP levels during acute infection. The low phenotypic score of *Tg*PDE16 and *Tg*PDE17 are rather unexpected because they are not expressed in tachyzoites; however, their transcriptional upregulation in the felid host hints a role during merogony and gametogenesis. Similarly, *Tg*PDE5 and *Tg*PDE6 may be required during chronic infection.

Intriguingly, all parasite PDEs were predicted to be membrane-associated proteins, and most of them lack a defined regulatory domain. Out of 4 PDEs harboring an ancillary module, *Tg*PDE2 comprises a GAF domain, which is generally a hallmark of cGMP and dual-specific human PDEs and involved in allosteric control of the catalytic activity [43]. *Tg*PDE4 contains an ion transport domain resembling cation channels – an apparently exclusive feature – that implies a coordination of cyclic nucleotide signaling with cation transport. Likewise, *Tg*PDE15 and *Tg*PDE18 harbor OCRE and MAEBL motifs, respectively. While the function of former module is quite elusive, the MAEBL protein in *Plasmodium* was shown as essential for sporozoite infection of both the mosquito salivary gland and the mammalian hepatocytes [60], [61], [62]. Among the parasite PDEs with no second domain, we observed that *Tg*PDE3 and *Tg*PDE14 grouped together in our phylogenetic analysis, showed identical substrate specificity, and are likely expressed during sexual development. Strikingly though, *Tg*PDE14 has a partial deletion in the catalytic region (Fig. S1), questioning its enzymatic activity while reasoning its functional substitution by *Tg*PDE3. Contrariwise, *Tg*PDE8 and *Tg*PDE9 share the same subcellular location, tertiary topology and substrate specificity, and the latter is not needed for the lytic cycle. Therefore, the two proteins may be functionally-redundant *twin* PDEs in tachyzoites of *T. gondii*.

Selective inhibition of cyclic nucleotide PDEs has long been of interest as promising therapeutic targets against several diseases including central nervous, immune and cardiovascular system disorders, infertility and metabolic malfunctions [42]. In context of our work, the PDE8 (cAMP-specific) and PDE9 (cGMP-specific) protein families are characterized as insensitive to non-selective PDE-inhibitor IBMX [63], while their isoforms can be potently inhibited by PF-04957325 [63], [64] and BIPPO [39], [65], respectively. BIPPO has also been shown to inhibit cGMP-specific *Pf*PDEα [39], and commonly used to modulate cGMP-dependent events in *T. gondii* tachyzoites [13], [18], [25], [26]. This inhibitor was recently demonstrated to be effective against dual-specific *Pf*PDEβ [35], implying its possible effect on cAMP- as well as cGMP- dependent processes [2], [13], [65]. Our results suggest that *Tg*PDE9 is likely not a target of BIPPO and IBMX even at higher concentrations used herein (50–100 μM), but can be partially inhibited by a much lower amount of PF-04957325 (10 μM). The latter drug may also inhibit other cAMP and dual-specific parasite PDEs, and its druggability in culture remains to be tested. Going forward, our smHA-tagged strains should enable the isolation of selected PDEs for their catalytic and drug-inhibition studies.

## 4 Material and methods

### 4.1 Biological reagents

Human foreskin fibroblast (HFF) cells were provided by Carsten Lüder (Georg-August University, Göttingen, Germany). The RHΔ*ku80-hxgprt*^*-*^ strain of *T. gondii* was obtained from Vern Carruthers (University of Michigan, Ann Arbor, USA). The parasite strain lacks the non-homologous end-joining repair and hypoxanthine-xanthine-guanine phosphoribosyltransferase (HXGPRT) expression, and thereby permits efficient homologous recombination and positive transgenic selection for genome editing [66], [67], [68]. The *pU6-Cas9-UPRT_sgRNA_* for expression of Cas9 and *single guide* RNA (*sg*RNA), as well as *pTUB1-YFP-mAID-3xHA-HXGPRT* plasmids were provided by David Sibley (Washington University in St. Louis, USA). The *pLIC-smHA-CAT* vector harboring the *spaghetti monster* HA (smHA or 10xHA) epitope [69] was offered by Bang Shen (Huazhong Agricultural University, Wuhan, China). The *pTUB1-YFP-smHA-HXGPRT* plasmid, used here for 3′-genomic tagging of PDEs, was constructed from *pTUB1-YFP-mAID-3xHA-HXGPRT* by excising the mAID-3xHA region (*Nhe*I and *Nde*I digestion), followed by cloning of smHA amplicon. The latter was amplified from *pLIC-smHA-CAT* vector using specific primers containing the *Nhe*I and *Nde*I restriction sites (Table S2). All steps of cloning were performed in *E. coli* (XL1B) strain.

The culture media and additives were purchased from PAN Biotech (Germany), and DNA-modifying enzymes were obtained from New England Biolabs (Germany). The reagent kits for cloning and isolation of nucleic acids were acquired from Life Technologies and Analytik Jena (Germany). The primary antibodies against *Tg*Gap45 [70], *Tg*Hsp90 [71], *Tg*Isp1 [54] and *Tg*Sag2 [72] proteins were provided by Dominique Soldati-Favre (University of Geneva, Switzerland), Sergio Angel (IIB-INTECH, Buenos Aires, Argentina), Peter Bradley (University of California, Los Angeles, USA) and Honglin Jia (Harbin Veterinary Research Institute, China), respectively. Other antibodies (mouse and rabbit) against HA-epitope were obtained from ThermoFisher Scientific (Germany) and Takara-Bio (Japan). The fluorophore-conjugated secondary antibodies (Alexa488, Alexa594; IRDye 680RD and 800CW) and oligonucleotides were acquired from Life Technologies (Germany). Other common laboratory chemicals were supplied by Sigma-Aldrich (Germany).

### 4.2 Host cell and parasite cultures

Tachyzoites were maintained in confluent HFF monolayers by infecting them every second day at a multiplicity of infection (MOI) of 3, as described elsewhere [73]. The HFF cultures were harvested by trypsinization and seeded into dishes or flasks as required. Uninfected as well as infected cultures were maintained in a humidified incubator (37 °C, 5% CO_2_) in Dulbecco’s Modified Eagle’s Medium (DMEM) containing glucose (4.5 g/L), heat-inactivated fetal bovine serum (10%), glutamine (2 mM), sodium pyruvate (1 mM), 1x minimum Eagle’s medium nonessential amino acids, penicillin (100 U/mL) and streptomycin (100 μg/mL). Parasites for all experiments were harvested by passing them through 27-gauge syringe (2x) unless specified otherwise.

### 4.3 Epitope-tagging of *Tg*PDE1-18 in tachyzoites

To determine the expression and subcellular localization, 3′-insertional tagging (3′IT) of *Tg*PDE1-18 genes with smHA-tag was performed. Sequences of all parasite PDEs were retrieved from ToxoDB [40]. Oligonucleotides encoding gene-specific *sg*RNA to target the 3′-end (≈20 nucleotides upstream of the stop codon) were designed using the Eukaryotic Pathogen CRISPR Guide RNA/DNA Design Tool (grna.ctegd.uga.edu). The *pU6-Cas9-TgPDEx_sgRNA_* (x = 1–18) constructs expressing Cas9 and *sg*RNA of respective PDEs were generated by Q5 site-directed mutagenesis (New England Biolabs). The PCR amplicons harboring smHA and HXGPRT selection cassette (HXGPRT S. C.) flanked by 5′ and 3′ homology arms of individual PDEs were generated by the Phanta Max Super-Fidelity DNA polymerase (Vazyme Biotech, China) using *pTUB1-YFP-smHA-HXGPRT* template. For all PDEs, the 5'-homology arm contained 50 nucleotides preceding the stop codon, while the 3'-homology arm included 50 nucleotides downstream of the *sg*RNA target region of corresponding PDEs (Table S2).

To make transgenic strains, extracellular tachyzoites (RHΔ*ku80-hxgprt^-^*, 1-2 × 10^7^) were pelleted by centrifugation (420*g*, 10 min), followed by resuspension in 100 μL of filter-sterile cytomix (120 mM KCl, 0.15 mM CaCl_2_, 10 mM K_2_HPO_4_/KH_2_PO_4_, 25 mM HEPES, 2 mM EGTA, 5 mM MgCl_2_, pH 7.6) supplemented with fresh ATP (2 μM) and glutathione (5 μM). Independent batches of tachyzoites were electroporated with the purified PCR amplicon (2 μg) and *sg*RNA construct of individual PDEs (15 μg) according to manufacturer’s protocol (Amaxa, T-016 program, 1700 V, 50 Ω, 2 pulses of 176 μs at the interval of 100 ms, unipolar, Lonza Group AG). Transgenic parasites expressing HXGPRT were positively selected by mycophenolic acid (25 μg/mL) and xanthine (50 μg/mL) [68]. The tagging of PDEs was confirmed by genomic screening using recombination-specific primers and subsequent sequencing of amplicons. Clonal strains were isolated by limiting dilution of drug-resistant transgenic parasites in 96-well plates. Expression of smHA-tagged PDEs in the eventual clonal strains (P_native_-*Tg*PDEx-smHA_3′IT_-HXGPRT_3′UTR_, x = 1–18) was driven by the native promoter and HXGPRT-3′UTR. However, a possibility of protein mislocalization due to the fusion with smHA-tag cannot be excluded.

### 4.4 Immunofluorescence assay

To stain intracellular tachyzoites, confluent HFF cells grown on glass coverslip in 24-well plates were infected with indicated strains (MOI, 3). Cultures (24–30 h infection) were washed with PBS (500 μL), fixed with 4% paraformaldehyde (10 min) and neutralized with 0.1 M glycine/PBS (5 min). Afterward, cells were permeabilized in 0.2% Triton X-100/PBS (20 min), and blocked in 2% BSA/0.2% Triton X-100/PBS (20 min) to reduce non-specific immunostaining. Samples were stained with the primary antibodies in the blocking solution (mouse α-HA, 1:3000 and rabbit α-*Tg*Gap45, 1:8000; or rabbit α-HA, 1:3000 and mouse α-*Tg*Isp1, 1:2000; 1 h), followed by 3x washes (5 min each) with 0.2% Triton X-100/PBS. Cells were finally treated with fluorophore-conjugated secondary antibodies (Alexa488, 1:4000 and Alexa594, 1:4000) diluted in 2% BSA/0.2% Triton X-100/PBS (45 min). Following three additional PBS washes, coverslips were mounted in Fluoromount G/DAPI solution and stored in dark at 4 °C for microscopy. To induce the splitting of the IMC and PM, extracellular parasites (10^5^) were treated with α-toxin from *Clostridium septicum* (20 nM, 3 h, 37 °C; List Biological Laboratories, USA). Tachyzoites were then released on the BSA-coated (0.01% in PBS) coverslips, allowed to settle for 20 min, fixed with 4% paraformaldehyde containing 0.05% glutaraldehyde and immunostained with α-*Tg*Gap45 (1:8000), α-*Tg*Sag2 (1:1000) and/or α-HA (1:10000), as reported [25]. Extracellular as well as intracellular parasites were imaged by an ApoTome microscope (Zeiss, Germany).

### 4.5 Immunoblot analysis

Fresh tachyzoites (6 x 10^7^) were harvested from parasitized cultures, washed once with ice-cold PBS and pelleted (8000*g*, 3 min, 4 °C). Parasites were lysed in 55 μL buffer (10 mM K_2_HPO_4_, 150 mM NaCl, 5 mM EDTA, 5 mM EGTA, pH7.4; 0.2% sodium deoxycholate, 1% Triton X-100) containing freshly-added protease inhibitors (trypsin, 20 µg/mL; aprotinin, 10 µg/mL; benzamidin, 500 µg/mL; PMSF, 0.5 mM; Na_3_VO_4_, 0.1 mM; NaF, 50 mM). Samples were incubated on ice for 30 min and then centrifuged (20000*g*, 15 min, 4 °C) to collect the cell free extract (50 μL), which were mixed with 5× loading buffer (13 μL, no boiling), followed by SDS-PAGE (6–8%). Proteins were blotted onto a nitrocellulose membrane (85 mA/cm^2^, 100 min, semidry), and stained overnight (4 °C) with mouse α-HA (1:10000) and rabbit α-*Tg*Hsp90 (1:10000) antibodies diluted in 5% skimmed milk solution with 0.2% Tween 20/TBS. Immunoblot was washed 3x with 0.2% Tween 20/TBS (5 min) and incubated with IRDye-conjugated secondary antibodies (680RD and 800CW, 1:15000) for 1 h. The protein bands were visualized using an Odyssey Fc imaging system (LI-COR Biosciences).

### 4.6 Immunoprecipitation and PDE assays

The pull-down of native *Tg*PDE8-smHA_3′IT_ and *Tg*PDE9-smHA_3′IT_ proteins were performed by using the monoclonal α-HA agarose (clone HA-7, Sigma Aldrich, Germany). Cell free extract was prepared as reported in “*immunoblot analysis*”, mixed with 25–50 μL of agarose beads, and the volume was adjusted to 1 mL by adding lysis buffer supplemented with protease inhibitor cocktail (*see above*). Subsequent to incubation with constant shaking (6 h, 4 °C), beads were pelleted (200*g*, 30 s), washed once with ice-cold lysis buffer containing protease inhibitor cocktail and then twice with distilled H_2_O to eliminate any possible phosphate contamination. The immunoprecipitated protein pellets (*Tg*PDE8-smHA_3’IT_ and *Tg*PDE9-smHA_3’IT_) bound to agarose beads were tested by a colorimetric phosphodiesterase assay (Abcam, ab139460). Briefly, beads resuspended in the assay buffer were distributed into wells of a microplate. The substrate (cAMP or cGMP, 200 μM) was added, immediately followed by the supplement of 5’-nucleotidase to release the phosphate group of nucleotide monophosphate (AMP or GMP). The reaction was performed at 37 °C (1 h) and terminated by the addition of 100 μL green assay reagent (30 min, room temperature); subsequently the OD_620_ was measured. The cAMP-specific PDE from bovine brain was included as a positive control alongside, as described by the kit manufacturer. 3-isobutyl-1-methylxanthine (IBMX), 5-benzyl-3-isopropyl-1H-pyrazolo [4,3d] pyrimidin-7(6H)-one (BIPPO) [39] and PF-04957325 (Pfizer, Inc) were also tested for their inhibitory effect on *Tg*PDE9. Standards with varying phosphate concentrations (0.25–4 nmol) were utilized to quantify the enzymatic hydrolysis of cAMP and cGMP.

### 4.7 Genetic deletion of *Tg*PDE9 and plaque assays

*Tg*PDE9 gene was deleted by double homologous crossover in the *Tg*PDE9-smHA_3′IT_ strain. A dual CRISPR/Cas9 vector expressing two *sg*RNA targeting the 5′UTR region of *Tg*PDE9 and HXGPRT expression cassette was designed (Fig. 7C). To achieve this, locus-specific CRISPR plasmids were generated by replacing the *UPRT*-targeting *sg*RNA in the *pSAG1-Cas9-sgUPRT* vector by Q5 site-directed mutagenesis (New England Biolabs), and then fused to make a knockout construct (Table S2). In brief, amplicon containing the U6-*sg*HXGPRT (678 bp) was amplified using *pSAG1-Cas9-sgHXGPRT* template and respective primers (HXGPRT-*sg*RNA-*Kpn*I-F/HXGPRT-*sg*RNA-*Xho*I-R), and cloned into *Kpn*I-*Xho*I-digested *pSAG1-Cas9-sgTgPDE9-5′UTR* vector. The dual CRISPR plasmid was transfected along with a donor amplicon, which comprised of a DHFR-TS selection cassette flanked by 5′- and 3′-homology arms (70 bp each) targeting the upstream and downstream regions of *Tg*PDE9 gene (see scheme in Fig. 7C). Stable transgenic parasites were selected using 1 μM pyrimethamine as reported elsewhere [74] and cloned by limiting dilution. To perform plaque assays, confluent HFF monolayers grown in 6-well plates were infected with 150 parasites/well and incubated for 7 days (37 °C, 5% CO_2_) in standard culture medium without any perturbation. Samples were washed twice with PBS, fixed with ice-cold methanol (10 min), stained with crystal violet (15 min) and imaged.

### 4.8 Sequence analysis and phylogenetic clading

The PDE sequences of chosen protozoan and metazoan organisms were acquired from the UniprotKB (www.uniprot.org) or Eukaryotic Pathogen Database (www.EuPathDB.org) [75]. Information from different tools was utilized to schematize the primary structures of *Tg*PDE1-18 by Illustrator for Biological Sciences v1.0.3 [76]. The protein coding sequences were examined by TMHMM [77], Phobius [78] and TMpred [79] programs to deduce the number, size, orientation and location of the transmembrane helices. Only those TMs with a confidence score ≥ 670 were considered to discern the membrane topology. The NCBI conserved domain search tool [80], SMART [81] and PFAM v32.0 [82] were deployed to assess the PDEase I and other auxiliary domains. The secondary structure of selected PDEs having an ancillary domain were constructed using TeXtopo package (v1.4) in LaTeX typesetting system [83]. Sequences were aligned by ClustalW (gap opening penalty, 10.00) algorithm, and the Neighbor-Joining method was applied for an estimated pairwise distance matrix. Phylogeny of the whole sequence and catalytic domains of 105 PDEs was performed using the Maximum Likelihood method and JTT matrix model for amino acid substitution in MEGA-X software [84]. The cladograms were visualized by Interactive Tree of Life (iTOL) program [85].

### 4.9 Prediction of catalytic specificity and structure modeling

Human PDEs grouped according to their substrate specificity (*Hs*PDE1, 2, 3, 10 and 11 as dual-specific; *Hs*PDE4, 7 and 8 as cAMP-specific; *Hs*PDE5, 6, and 9 as cGMP-specific) were aligned in ClustalW to obtain three consensus reference sequences corresponding to their respective catalytic activities. These *substrate-specific* sequences of human PDEs were clustered with the catalytic domain of each parasite PDE using the Maximum Likelihood method (MEGA-X, [84]). The approach was tested with well-described PDE sequences of *H. sapiens*, *D. melanogaster*, *C. elegans* and *P. falciparum*. The accuracy of prediction was *circa* 80% with metazoan PDEs and 50% for *Plasmodium* PDEs. We also analyzed the clading patterns of 4 *Pf*PDEs with *Tg*PDE1-18 to consolidate our data. Sequence identity and similarity of *Tg*PDE1-18 were calculated by aligning their catalytic domain sequences in the SIAS program using default parameters (http://imed.med.ucm.es/Tools/sias.html).

Three dimensional models for the catalytic region of *Tg*PDE9 (Ser802–Ser1113) and *Tg*PDE8 (Leu623-Val937) were generated based on substrate-bound structures of *Hs*PDE4D (PDB, 2pw3 [57]) and *Hs*PDE9A (PDB, 3dyn [56]) using SWISS-MODEL suite [86]. The Global Model Quality Estimation (GMQE) scores of *Tg*PDE9 models, which reflect the accuracy of homology models, were determined as 0.69 (2pw3-based model) and 0.68 (3dyn-based model), and the corresponding Quality Model Energy Analysis (QMEAN) scores were −2.84 and −2.81, respectively. Similarly, the GMQE values for *Tg*PDE8 modeled on the 2pw3 and 3dyn crystal structures were determined as 0.72 and 0.71, and the corresponding QMEANs were scored as −2.45 and −2.05, respectively. The substrate docking was performed using the AutoDock Vina plugin [87] in CHIMERA software [88]. cAMP and cGMP were positioned into the catalytic pockets of *Tg*PDE8 or *Tg*PDE9 model following their respective location in *Hs*PDE4D and *Hs*PDE9A structures. Equally, the binding of *Tg*PDE8 and *Tg*PDE9 with PDE-inhibitors, BIPPO and PF-04957325, was tested by docking of their chemical structures within the catalytic region.

## Declaration of Competing Interest

The authors declare that they have no known competing financial interests or personal relationships that could have appeared to influence the work reported in this paper.
