# Supplementary material for: The protozoan parasite Toxoplasma gondii encodes a gamut of phosphodiesterases during its lytic cycle in human cells
Source: Comput Struct Biotechnol J. 2020 Nov 21;18:3861–76. doi: 10.1016/j.csbj.2020.11.024 (PMC7720076; doi:10.1016/j.csbj.2020.11.024)
Supplement: Supplementary data 1 [file mmc1.pdf]

SUPPLEMENT FIGURE LEGENDS

Fig S1: *TgPDE1-18 alignment with substrate-specific consensus sequences of human PDEs.* Of 21 human PDEs, the groups of cAMP- (PDE4, 7, 8), cGMP- (PDE5, 6, 9) and dual- (PDE1, 2, 3, 10, 11) specific PDEs were separately aligned to obtain consensus sequences. The PDE signature residues broadly well-conserved across *Tg*PDE1-18 are marked by inverted yellow triangles, whereas three (somewhat) divergent residues are labeled by upright green triangles. The stretches of low homology region in *Tg*PDE17 and 18 were trimmed to improve the visualization. Also note that *Tg*PDE4 shows a high deviation from the signature amino acids, and *Tg*PDE14 contains a deletion in the catalytic region. The blue–red gradient scale features the conservation of residues throughout PDEs.

Fig S2: *Membrane topology of Toxoplasma PDEs containing a secondary domain*. The color-coded illustrations of indicated PDEs highlighting the conserved domains were made using TeXtopo (v1.4) program. The portrayed topology is built on the number of TM helices predicted herein (Figure 1).

Fig S3: A cladogram of the full-length *Tg*PDE1-18 proteins with their orthologs from *Homo sapiens* (*Hs*), *Drosophila melanogaster* (*Dm*), *Danio rerio* (*Dr*), *Caenorhabditis elegans (Ce*), *Dictyostelium discoideum* (*Dd*), *Cavenderia fasciculata* (*Cf*), *Leishmania major* (*Lm*), *Trypanosoma brucei* (*Tb*), *Eimeria tenella* (*Et*) and *Plasmodium falciparum* (*Pf*). The clading analysis was performed using the Maximum Likelihood method (1000 bootstraps, gray spheres). The UniprotKB accession numbers along with organism abbreviations (in bracket) are indicated except for the human and apicomplexan PDEs (see Table S1; *Tg*, red; *Et,* blue; *Pf,* green). A comparable cladogram of the catalytic domains of represented PDEs can be seen in Figure 2A.

Fig S4: Identity **(A)** and similarity **(B)** matrix of the catalytic regions of *Tg*PDE1-18. Homology levels of PDE enzymes are depicted in percentage to reflect their evolutionary relatedness.

Fig S5: *Immunostaining of TgPDE5, TgPDE8 and TgPDE18, and* α*-toxin-induced splitting of plasma membrane and inner membrane complex in T. gondii.* **(A)** Intracellular tachyzoites expressing smHA-tagged PDE proteins (30 h infection) were stained with α-HA and α-*Tg*Gap45 antibodies. Nuclei were visualized by DAPI (scale, 20 μm). Images were obtained using transgenic strains as described in Figure 3A. Note that not all parasitophorous vacuoles in the designated strains were stained for HA. **(B)** α-toxin treatment of extracellular tachyzoites. The parasite strains expressing specified smHA-tagged PDEs were treated with the drug to disband the inner membrane complex and plasmalemma and then immunostained. The parasite nuclei were stained using DAPI (scale, 2 μm).

Fig S6: The topology of *Tg*PDE8 and docking of substrates and inhibitors. **(A-B)** Homology models of *Tg*PDE8 superimposed with the structure of *Hs*PDE9A (PDB, 3dyn) **(A)** *Hs*PDE4D (PDB, 2pw3) **(B).** Human PDEs are shown in gray and corresponding 3D models of the *Tg*PDE8 catalytic domain are in salmon and blue. Metal ions, Zn^{2+} (purple) and Mg^{2+} (green) bound to the catalytic center are also shown. **(C-D)** Substrate-binding region of *Tg*PDE8 in cGMP- **(C)** and cAMP- **(D)** bound states, as deduced from the homology models based on *Hs*PDE9A and *Hs*PDE4D respectively. **(E)** Docking of BIPPO into *Tg*PDE8 model (salmon) overlaid with cGMP-bound *Hs*PDE9A (gray). **(F)** Simulation of PF-04957325 interaction with *Tg*PDE8 (blue) superimposed with cAMP-bound *Hs*PDE4D (gray).

Fig S7: *The catalytic region of TgPDE8 and TgPDE9 aligned to HsPDE4D and HsPDE9A.* Selected residues are marked with color-coded triangles to specify their importance for the phosphodiesterase activity. Alpha-helices are depicted following the tertiary models in Figure 6 and S6. The numbering of human PDEs, as indicated, is based on the position of glutamine (Q) switch reported in the crystal structures of *Hs*PDE4D (Q369) and *Hs*PDE9A (Q453). The sequence conservation is reflected by a blue-red gradient.

Figure S1

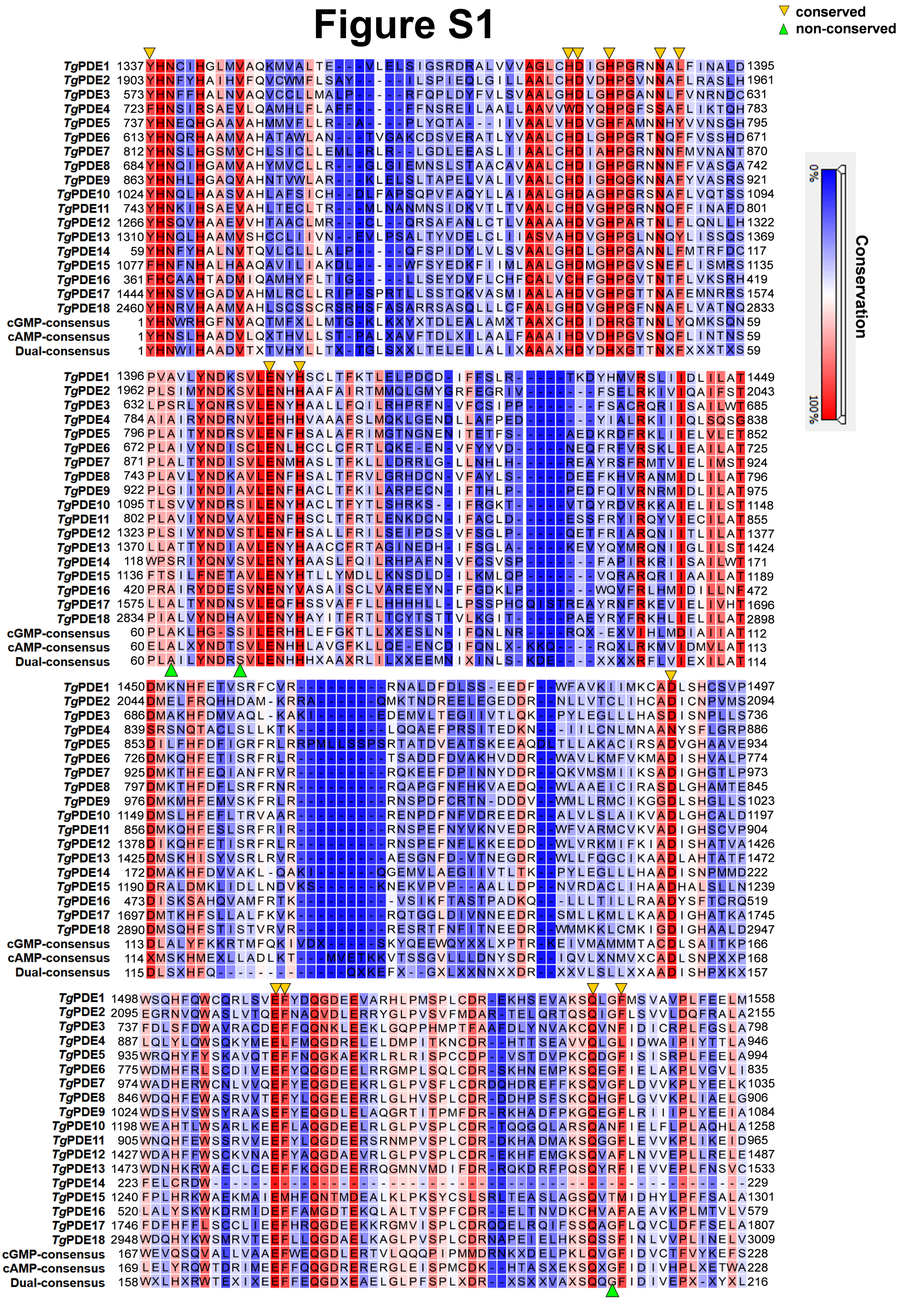

conserved
non-conserved
Conservation
0%
100%
TgPDE1 1337 YHNCIHGLMVAQKMVALTE---VLELSIGSRDRALVVVAGLCHDIGHPGRNNALFINALD 1395
TgPDE2 1903 YHNFYHAIHVFQVCWMFLSAY----ILSPIEQLGILLAALCHDVDHPGVNNAFLRASLH 1961
TgPDE3 573 YHNFFHALNVAQVCCLLMALP----RFQPLDYFVLSVAALGHDLGHPGANNLFVNRNDC 631
TgPDE4 723 FHNSIRSAEVLQAMHLFLAFF----FFNSREILAALLAAVVWDYQHPGFSSAFLIKTQH 783
TgPDE5 737 YHNEQHGAAVAHMMVFLLR--A---PLYQTA---IIVAALVHDVGHFAMNNHYVVNSGH 795
TgPDE6 613 YHNQRHAAMVAHATAWLAN---TVGAKCDSVERATLYVAALCHDLGHPGRTNQFFVSSHD 671
TgPDE7 812 YHNSLHGSMVCHLSICLLEML--RLR--LGDLEEASLIIAALCHDIAHPGRNNNFMVNANT 870
TgPDE8 684 YHNQIHGAMVAHYMVCLLR---GLGIEMNSLSTAACAVAALCHDIGHPGRNNNFFVASGA 742
TgPDE9 863 YHNHLHGAQVAHNTVWLAR---KLELSLTAPELVALIVAALCHDIGHQGKNNAFYVASRS 921
TgPDE10 1024 YHNQLHAASVAHLAFSICH---DLFAPSQPVFAQYLLAIAALGHDAGHPGRSNAFLVQTSS 1094
TgPDE11 743 YHNKIHSAEVAHLTECLTR---MLNANMNSIDKVTLTVAALCHDVGHPGRNNQFFINAFD 801
TgPDE12 1266 YHSQVHAAEVVHTAACLMR---CL--QRSAFANLCTLVAAAAHDVGHPARTNLFLQNLLH 1322
TgPDE13 1310 YHNQLHAAMVSHCCLIIVN---EVLPSALTYVDELCLIASVAHDVGHPGLNNQYLISSQS 1369
TgPDE14 59 YHNFYHALNVTQVLCLLALP----QFSPIDYLVLSVAALGHDLGHPGANNLFMTRFDC 117
TgPDE15 1077 FHNFNHALHAAQAVILIAKDL----WFSYEDKFIIMLAALGHDMGHPGVSNEFLISMRS 1135
TgPDE16 361 FHCAAHTADMIQAMHYFLTIG----LLSEYDVFLCHFCALVCHFGHPGVTNTFLVKSRH 419
TgPDE17 1444 YHNSVHGADVAHMLRCLLRIP-SPRTLLSSTQKVASMIAALAHDVGHPGTTNAFEMNRRS 1574
TgPDE18 2460 YHNRVHAAMVAHLSCSSCRSRHSFASARRSASQLLLCFAALGHDVGHPGFNNAFLVATNQ 2833
cGMP-consensus 1 YHNWRHGFNVAQTMFXLLMTG-KLKXYXTDLEALAMXTAAXCHDIDHRGTNNLYQMKSQN 59
cAMP-consensus 1 YHNSLHAADVLQXTHVLLST-PALXAVFTDLXILAALFAAAIHDVDHPGVSNQFLINTNS 59
Dual-consensus 1 YHNWIHAADVTXTVHYLLTX-TGLSXXLTELEIXLALIXAAAXHDYDHXGTTNXFXXXTXS 59
TgPDE1 1396 PVAVLYNDKSVLENYHSCLTFKTLELPDCD-IFFSLR-----TKDYHMVRSLIIDLILAT 1449
TgPDE2 1962 PLSIMYNDKSVLENHHAAFAIRTMMQLGMYGRFEGRIV-------FSELRKIVIQAIFST 2043
TgPDE3 632 LPSRLYQNRSVLENYHAALLFQILRHPRFN-VFCSIPP-------FSACRQRIISAILWT 685
TgPDE4 784 AIAIRYNDRNVLEHHHVAAAFSLMQKLGENDLLAFPED------YIALRKIIIQLSQSG 838
TgPDE5 796 PLAITYNDRSVLENFHSALAFRIMGTNGNENITETFS-----AEDKRDFRKLIIELVLET 852
TgPDE6 672 PLAIVYNDISCLENLHCCLCFRTLQKE-EN-VFYYVD-----NEQFRFVRSKLIEAILAT 725
TgPDE7 871 PLALTYNDISVLENMHASLTFKLLDRRLG-LLNHLH-----REAYRSFRMTVIELIMST 924
TgPDE8 743 PLAVLYNDKAVLENFHSALTFRVLGRHDCN-VFAYLS-----DEEFKHVRANMIDLILAT 796
TgPDE9 922 PLGIIYNDIAVLENFHACLTFKILARPECN-IFTHLP-----PEDFQIVRNRMIDLILAT 975
TgPDE10 1095 TLSVVYNDRSILENYHACLTFYTLSHRKS-IFRGKT-----VTQYRDVRKKAIELILST 1148
TgPDE11 802 PLAVIYNDVAVLENFHSCLTFRTLENKDCN-IFACLD-----ESSFRYIRQYVIECILAT 855
TgPDE12 1323 PLSIVYNDVSTLENFHSALLFRILSEIPDS-VFSGLP-----QETFRIARQNIITLILAT 1377
TgPDE13 1370 LLATTYNDIAVLENYHAACCFRTAGINEDH-IFSGLA-----KEVYQYMRQNIIGLILST 1424
TgPDE14 118 WPSRIYQNVSVLENYHAASLFQILRHPAFN-VFCSVSP-------FAPIRKRIISAILWT 171
TgPDE15 1136 FTSILFNETAVLENYHTLLYMDLLKNSDLD-ILKMLQP-------VQRARQRIIAAILAT 1189
TgPDE16 420 PRAIRYDDESVNENYVASAISCLVAREEYN-FFGDKLP-------WQVFRLHMIDILLNF 472
TgPDE17 1575 LLALTYNDNSVLEQFHSSVAFFLLHHHHLL-LPSSPHCQISTREAYRNFRKEVIELIVHT 1696
TgPDE18 2834 PIALVYNDHAVLENYHAYITFRTLTCYTSTTVLKGIT-----PAEYRYFRKHLIELILAT 2898
cGMP-consensus 60 PLAKLHG-SSILERHHLEFGKTLLXXESLN-IFQNLNR-----RQX-EXVIHLMDIAIIAT 112
cAMP-consensus 60 ELALXYNDTSVLENHHLAVGFKLLQE-ENCDIFQNLX-KKQ----RQXLRKMVIDMVLAT 113
Dual-consensus 60 PLAILYNDRSVLENHHHXAAXRLILXXEEMN-IXINLS-KDE----XXXXRFLVIEXILAT 114
TgPDE1 1450 DMKNHFETVSRFCVR----------RNALDFDLSS-EEDF--WFAVKIIMKCADLSHCSVP 1497
TgPDE2 2044 DMELFRQHHDAM-KRRA------QMKTNDREELEGEDDR--NLLVTCLIHCADICNPVMS 2094
TgPDE3 686 DMAKHFDMVAQL-KAKI------EDEMVLTEGIIVTLQK--PYLEGLLLHASDISNPLLS 736
TgPDE4 839 SRSNQTACLSLLKTK---------LQQAEFPRSITEDKN--IIILCNLMNAANYSFLGRP 886
TgPDE5 853 DILFHFDFIGRFRLRRPMLLSSPSRTATDVEATSKEEAQDLTLLAKACIRSADVGHAAVE 934
TgPDE6 726 DMKQHFETISRFRLR----------TSADDFDVAKHVDDR--WAVLKMFVKMADISHVALP 774
TgPDE7 925 DMKTHFEQIANFRVR----------RQKEEFDPINNYDDR--QKVMSMIIKSADIGHGTLP 973
TgPDE8 797 DMKTHFDFLSRFRNR----------RQAPGFNFHKVAEDQ--WLAAEICIRASDLGHAMTE 845
TgPDE9 976 DMKMHFEMVSKFRLR----------RNSPDFCRTN-DDDV--WMLLRMCIKGGDLSHGLLS 1023
TgPDE10 1149 DMSLHFEFLTRVAAR----------RENPDFNFVDREEDR--ALVLSLAIKVADLGHCALD 1197
TgPDE11 856 DMKQHFESLSRFRIR----------RNSPEFNYVKNVEDR--WFVARMCVKVADIGHSCVP 904
TgPDE12 1378 DIKQHFETISRFRLR----------RNSPEFNFLKKEEDD--WLVRKMIFKIADISHATVA 1426
TgPDE13 1425 DMSKHISYVSRLRVR----------AESGNFD-VTNEGDR--WLLFQGCIKAADLAHTATF 1472
TgPDE14 172 DMAKHFDVVAKL-QAKI------QGEMVLAEGIIVTLTK--PYLEGLLLHAADISNPMMD 222
TgPDE15 1190 DRALDMKLIDLLNDVKS------KNEKVPVP--AALLDP--NVRDACLIHAADHALSLLN 1239
TgPDE16 473 DISKSAHQVAMFRTK---------VSIKFTASTPADKQ--LLLTILLRAADYSFTCRQ 519
TgPDE17 1697 DMTKHFSLLALFKVK----------RQTGGLDIVNNEEDR--SMLLKMLLKAADIGHATKA 1745
TgPDE18 2890 DMSQHFSTISTVRVR----------RESRTFNFITNEEDR--WMMKKLCMKIGDIGHAALD 2947
cGMP-consensus 113 DLALYFKKRTMFQKIVDX-------SKYQEEWQYXXLXPTR-KEIVMAMMMTACDLSAITKP 166
cAMP-consensus 114 XMSKHMEXLLADLKT---MVETKKVTSSGVLLLDNYSDR--IQVLXNMVXCADLSNPXXP 168
Dual-consensus 115 DLSXHFQ-------------QXKEFX--GXLXXNNXXDR--XXVLSLLXXAADISHPXKX 157
TgPDE1 1498 WSQHFQWCQRLSVEEFYDQGDEEVARHLPMSPLCDR-EKHSEVAKSQLGFMSVAVPLFEELM 1558
TgPDE2 2095 EGRNVQWASLVTQEFNAQVDLERRYGLPVSVFMDAR-TELQRTQSQIGFLSVVLDQFRALA 2155
TgPDE3 737 FDLSFDWAVRACDEFFQQNKLEEKLGQPPHMPTFAAFDLYNVAKCQVNFIDICRPLFGSLA 798
TgPDE4 887 LQLYLQWSQKYMEELFMQGDRELELDMPITKNCDR--HTTSEAVVQLGLIDWAIPIYTTLA 946
TgPDE5 935 WRQHYFYSKAVQTEFFNQGKAEKRLRLRISPCCDP--VSTDVPKCQDGFISISRPLFEELA 994
TgPDE6 775 WDMHFRLSCDIVEEFYQQGDEELRRGMPLSQLCDR-SKHNEMPKSQEGFIELAKPLVGVLI 835
TgPDE7 974 WADHERWCNLVVQEFYEQGDEEKRLGLPVSFLCDRDQHDREFFKSQVGFLDVVKPLYEELK 1035
TgPDE8 846 WDQHFEWASRVVTEFYLQGEEERRLGLHVSPLCDR-DDHPSFSKCQHGFLGVVKPLIAELG 906
TgPDE9 1024 WDSHVSWSYRAASEFYEQGDLELAQGRTITPMFDR-RKHADFPKGQEGFLRIIIPLYEEIA 1084
TgPDE10 1198 WEAHTLWSARLKEEFLAQGDEELRLGLPVSPLCDR-TQQGQLARSQANFIELFLPLAQHLA 1258
TgPDE11 905 WNQHFEWSSRVVEEFYLQGDEERSRNMPVSPLCDR-DKHADMAKSQGGFLEVVKPLIKEID 965
TgPDE12 1427 WDAHFFWSCKVNAEFYAQGDAEVRLGLPVSPLCDR-EKHFEMGKSQVAFLNVVEPLLRELE 1487
TgPDE13 1473 WDNHKRWAECLCEEFFKQGDEERRQGMNVMDIFDR-RQKDRFPQSQYRFIEVVEPLFNSVC 1533
TgPDE14 223 FELCRDW--- 229
TgPDE15 1240 FPLHRKWAEKMAIEMHFQNTMDEALKLPKSYCSLSRLTEASLAGSQVTMIDHYLPFFSALA 1301
TgPDE16 520 LALYSKWKDRMIDEFFAMGDTEKQLALTVSPFCDR--ELTNVDKCHVAFAEAVKPLMTVLV 579
TgPDE17 1746 FDFHFFLSCCLIEEFHRQGDEEKKRGMVISPLCDRQQELRQIFSSQAGFLQVCLDFFSELA 1807
TgPDE18 2948 WDQHYKWSMRVTEEFLLQGDAELKAGLPVSPLCDRNAPEIELHKSQSSFINLVVPLINELV 3009
cGMP-consensus 167 WEVQSQVALLVAAEFWEQGDLERTVLQQQPIPMMDRNKXDELPKLQVGFIDVCTFVYKEFS 228
cAMP-consensus 169 LELYRQWTDRIMEEFFQQGDRERERGLEISPMCDK--HTASXEKSQXGFIDIVHPLXETWA 228
Dual-consensus 158 WXLHXRWTEXIXEEFFEQGDXEAELGLPFSPLXDR--XSXXVAXSQQGFIDIVEPX-XYXL 216

Figure S2

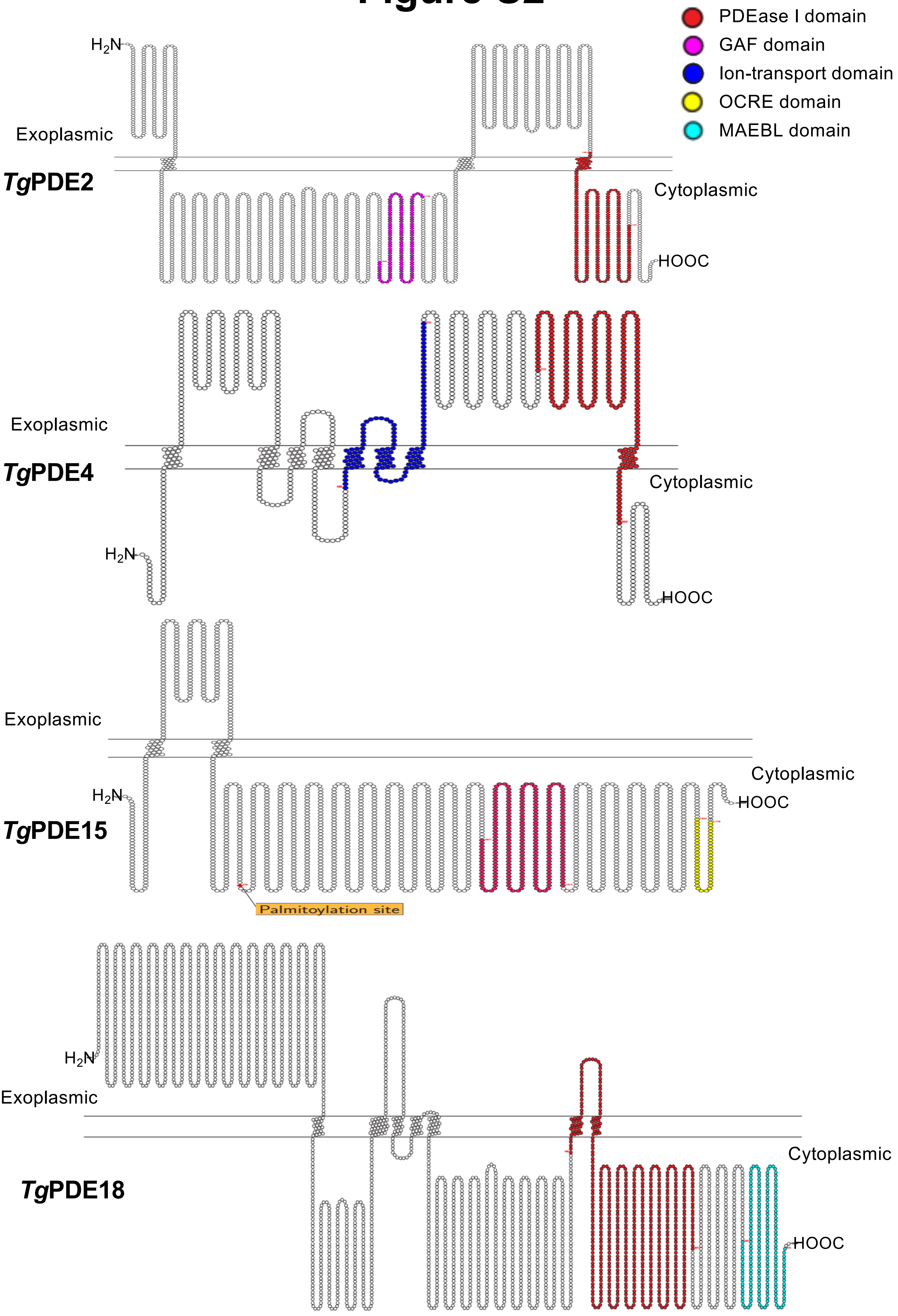

PDEase I domain
GAF domain
Ion-transport domain
OCRE domain
MAEBL domain
TgPDE2
Exoplasmic
Cytoplasmic
H_2N
HOOC
TgPDE4
Exoplasmic
Cytoplasmic
H_2N
HOOC
TgPDE15
Exoplasmic
Cytoplasmic
H_2N
HOOC
Palmitoylation site
TgPDE18
Exoplasmic
Cytoplasmic
H_2N
HOOC

Figure S3

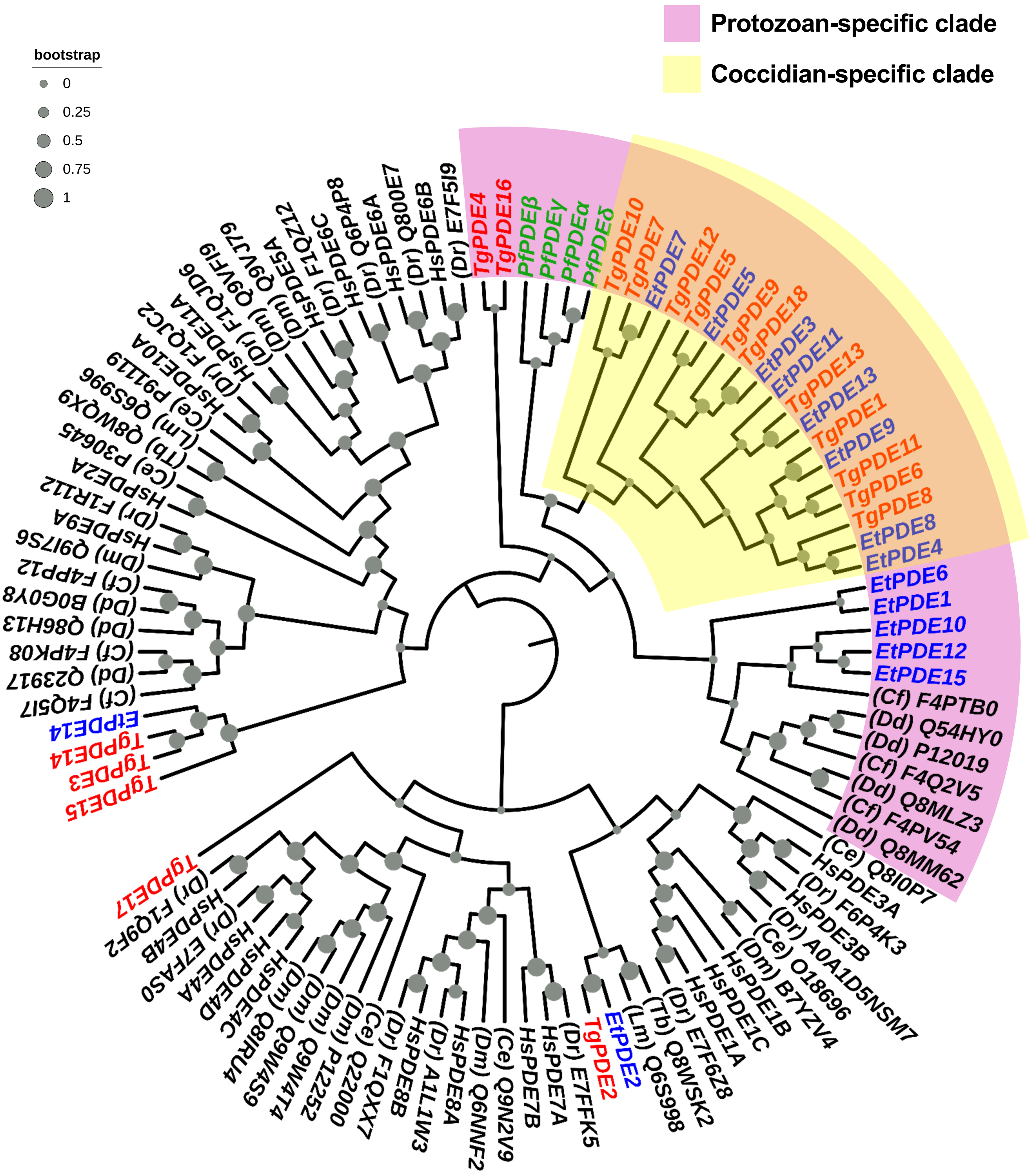
Protozoan-specific clade
Coccidian-specific clade
bootstrap
0
0.25
0.5
0.75
1
TgPDE4
TgPDE16
PfPDEβ
PfPDEγ
PfPDEα
PfPDEδ
TgPDE10
TgPDE7
EtPDE7
TgPDE12
TgPDE5
EtPDE5
TgPDE9
TgPDE18
EtPDE3
EtPDE11
TgPDE13
EtPDE13
TgPDE1
EtPDE9
TgPDE11
TgPDE6
TgPDE8
EtPDE8
EtPDE4
EtPDE6
EtPDE1
EtPDE10
EtPDE12
EtPDE15
(Cf) F4PTB0
(Dd) Q54HY0
(Dd) P12019
(Cf) F4Q2V5
(Dd) Q8MLZ3
(Cf) F4PV54
(Dd) Q8MM62
(Ce) Q8I0P7
HsPDE3A
(Dr) F6P4K3
HsPDE3B
(Dr) A0A1D5NSM7
(Ce) O18696
(Dm) B7YZV4
HsPDE1B
HsPDE1C
HsPDE1A
(Dr) E7F6Z8
(Tb) Q8WSK2
(Lm) Q6S998
EtPDE2
TgPDE2
(Dr) E7FFK5
HsPDE7A
HsPDE7B
(Ce) Q9N2V9
(Dm) Q6NNF2
HsPDE8A
(Dr) A1L1W3
HsPDE8B
(Dr) F1QXX7
(Ce) Q22000
(Dm) P12252
(Dm) Q9W4T4
(Dm) Q9W4S9
(Dm) Q8IRU4
HsPDE4C
HsPDE4D
HsPDE4A
(Dr) E7FAS0
HsPDE4B
(Dr) F1Q9F2
TgPDE17
TgPDE15
TgPDE3
TgPDE14
EtPDE14
(Cf) F4Q5I7
(Dd) Q23917
(Cf) F4PK08
(Dd) Q86H13
(Dd) B0G0Y8
(Cf) F4PP12
(Dm) Q9I7S6
HsPDE9A
(Dr) F1R112
HsPDE2A
(Ce) P30645
(Tb) Q8WQX9
(Lm) Q6S996
(Ce) P91119
HsPDE10A
(Dr) F1QJC2
HsPDE11A
(Dr) F1QJD6
(Dm) Q9VFI9
(Dm) Q9VJ79
(Dm) Q9VJ79
HsPDE5A
(Dr) F1QZ12
HsPDE6C
(Dr) Q6P4P8
HsPDE6A
(Dr) Q800E7
HsPDE6B
(Dr) E7F5I9

Figure S4

A Sequence Similarity

	TgPDE18	TgPDE17	TgPDE16	TgPDE15	TgPDE14	TgPDE13	TgPDE12	TgPDE11	TgPDE10	TgPDE9	TgPDE8	TgPDE7	TgPDE6	TgPDE5	TgPDE4	TgPDE3	TgPDE2	TgPDE1
TgPDE18	100	33.7	32.3	34.5	39.9	50.2	53.9	56.3	62.1	53	52.9	48.5	53.1	41.7	31.5	37.5	34.5	51.9
TgPDE17		100	36.2	32.8	38.2	47.6	50.4	49.6	48.3	47.9	47.5	55.3	49.6	42.1	35.3	35.8	32.6	47.2
TgPDE16			100	29.8	31.8	31.6	34.3	32.3	34	33.8	34.5	33.2	33.2	32.8	38.7	32.8	28.5	32
TgPDE15				100	42.8	32.5	32.2	32.8	34.9	33.8	29.8	31.5	33.2	30.7	28.6	42.4	33.2	33.3
TgPDE14					100	38.2	36.4	39.9	42.2	39.3	38.2	36.4	39.3	34.7	27.7	79.8	40.5	39.3
TgPDE13						100	47	51.1	47.6	48.9	49.8	49.4	51.3	44.2	32.9	36.4	37.7	42
TgPDE12							100	62.6	54.3	57	56.1	51.3	56.2	50	34.8	37.8	38.3	54.8
TgPDE11								100	52.5	59.8	65.4	57.9	66.8	49.2	34.5	37.5	36.3	63.2
TgPDE10									100	49.6	50.8	47.7	52.7	45.8	34.9	40.4	41.7	49.4
TgPDE9										100	58.1	54.7	56.6	50	35	39.3	37.2	59.3
TgPDE8											100	54.5	56.6	53.3	32.8	37.5	33.8	56.7
TgPDE7												100	54.9	48.9	33.6	37	40.4	56.7
TgPDE6													100	48.2	35	38.9	37.2	58.8
TgPDE5														100	34.5	35	33.3	50.2
TgPDE4															100	29.4	34.5	38.1
TgPDE3																100	37.5	38.5
TgPDE2																	100	39.4
TgPDE1																		100

B Sequence Identity

	TgPDE1	TgPDE2	TgPDE3	TgPDE4	TgPDE5	TgPDE6	TgPDE7	TgPDE8	TgPDE9	TgPDE10	TgPDE11	TgPDE12	TgPDE13	TgPDE14	TgPDE15	TgPDE16	TgPDE17	TgPDE18
TgPDE1	100																	
TgPDE2	28.6	100																
TgPDE3	28.1	28.7	100															
TgPDE4	25.5	24.4	20.6	100														
TgPDE5	37.7	25.8	26.7	23.1	100													
TgPDE6	46	30.1	29.2	23.9	35.8	100												
TgPDE7	43.3	31.1	26	23.4	35.3	44.7	100											
TgPDE8	48.9	28.3	28.3	21.4	44.2	46	45.5	100										
TgPDE9	45.9	27.8	30.3	21.8	38.5	46	43.2	47	100									
TgPDE10	39.4	31.3	28.7	24.4	33.8	44.2	40	38.8	38.5	100								
TgPDE11	53.2	28.7	25	22.3	36.7	54	44.7	55.4	47.4	42.5	100							
TgPDE12	45.2	32.6	28.3	21.7	40.9	48.7	42.6	48.3	42.6	42.6	53	100						
TgPDE13	34.2	29	27.3	22.5	33.8	40.3	36.8	38.5	36.8	39.8	39.8	37.8	100					
TgPDE14	26.6	33.5	76.9	19.1	24.3	28.3	24.3	26.6	28.3	29.5	27.2	27.7	26	100				
TgPDE15	23.8	27.3	34.5	22.3	21.4	22.1	22.6	21.4	23.9	26.5	23.1	24.3	23.8	32.4	100			
TgPDE16	23.8	21.7	23.8	26.8	25.1	24.3	24.7	26.8	23.9	23	21.7	25.7	23.4	22.5	20	100		
TgPDE17	35.1	25.1	25.4	25.2	31.4	36.7	46	35.8	34.6	36.7	38.3	38.3	34.6	26.6	23.5	26	100	
TgPDE18	39	27	28.7	22.3	31.7	43.8	41.7	42.5	38.5	52.5	44.6	44.8	40.7	30.1	24.8	25.5	25.7	100

Figure S5

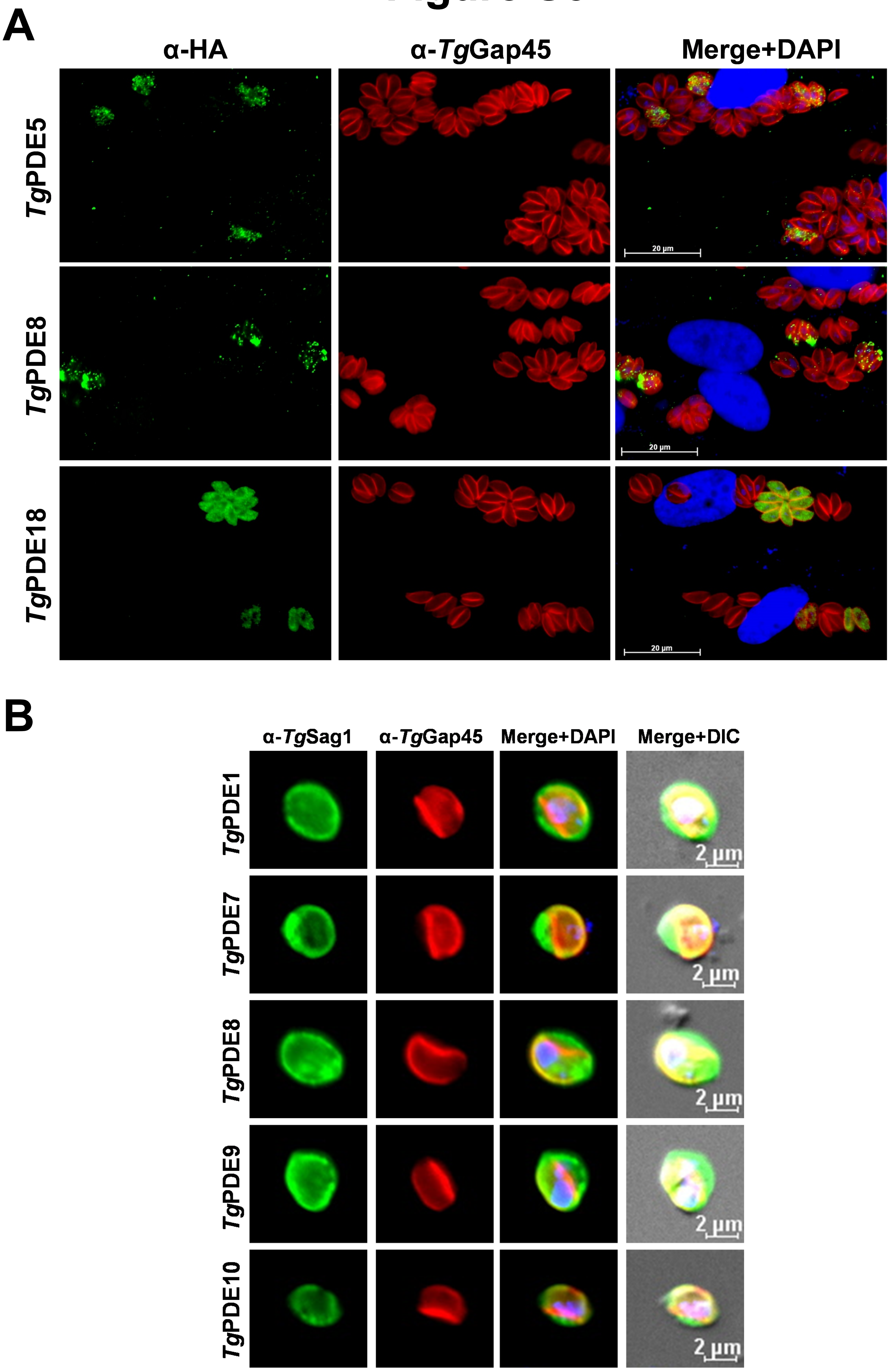
A
α-HA
α-TgGap45
Merge+DAPI
TgPDE5
TgPDE8
TgPDE18
20 µm
20 µm
20 µm
B
α-TgSag1
α-TgGap45
Merge+DAPI
Merge+DIC
TgPDE1
TgPDE7
TgPDE8
TgPDE9
TgPDE10
2 µm
2 µm
2 µm
2 µm
2 µm

Figure S6

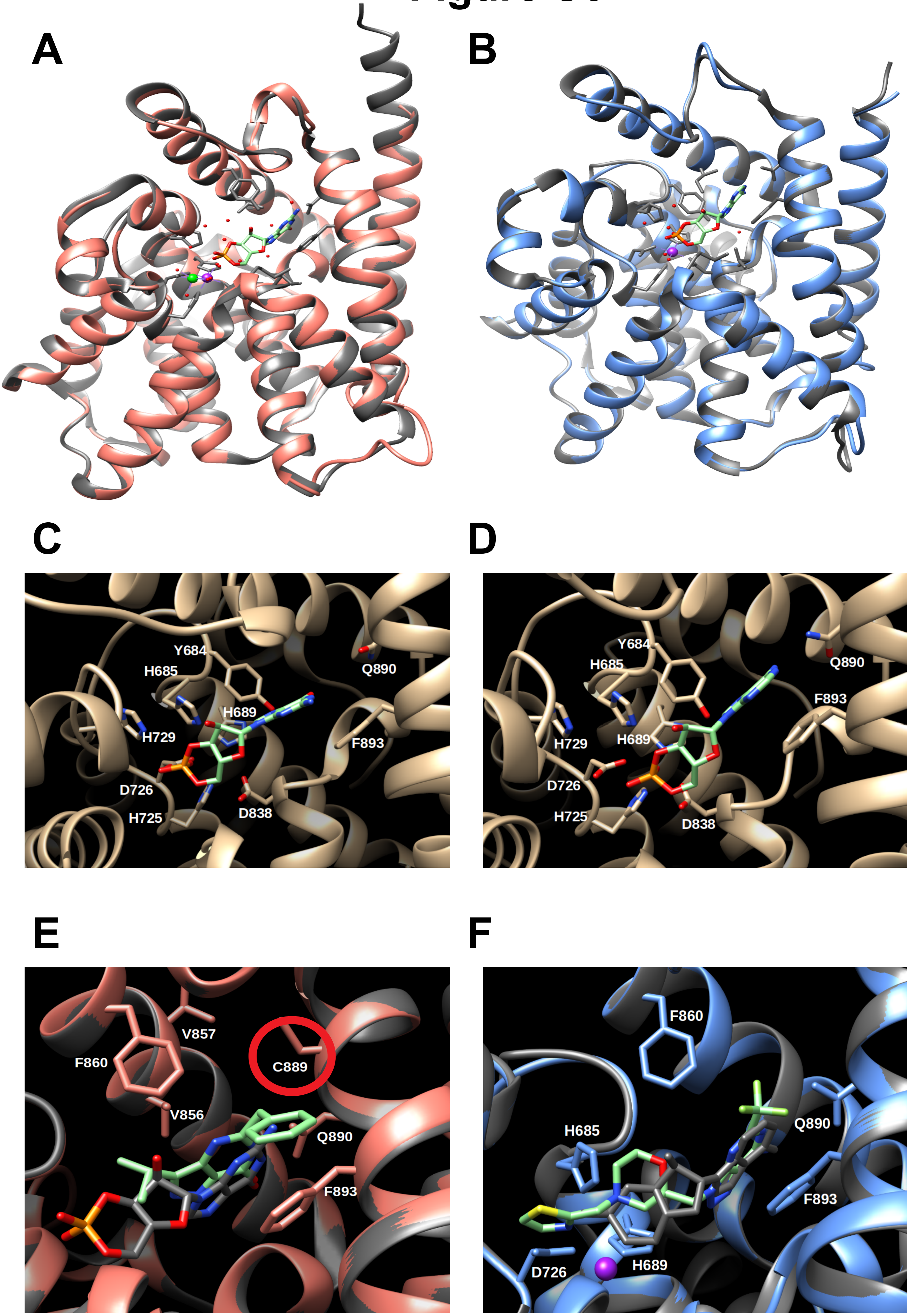
A
B
C
Y684
H685
H689
H729
D726
H725
D838
Q890
F893
D
Y684
H685
Q890
F893
H729
H689
D726
H725
D838
E
V857
F860
C889
V856
Q890
F893
F
F860
H685
Q890
F893
D726
H689

Figure S7

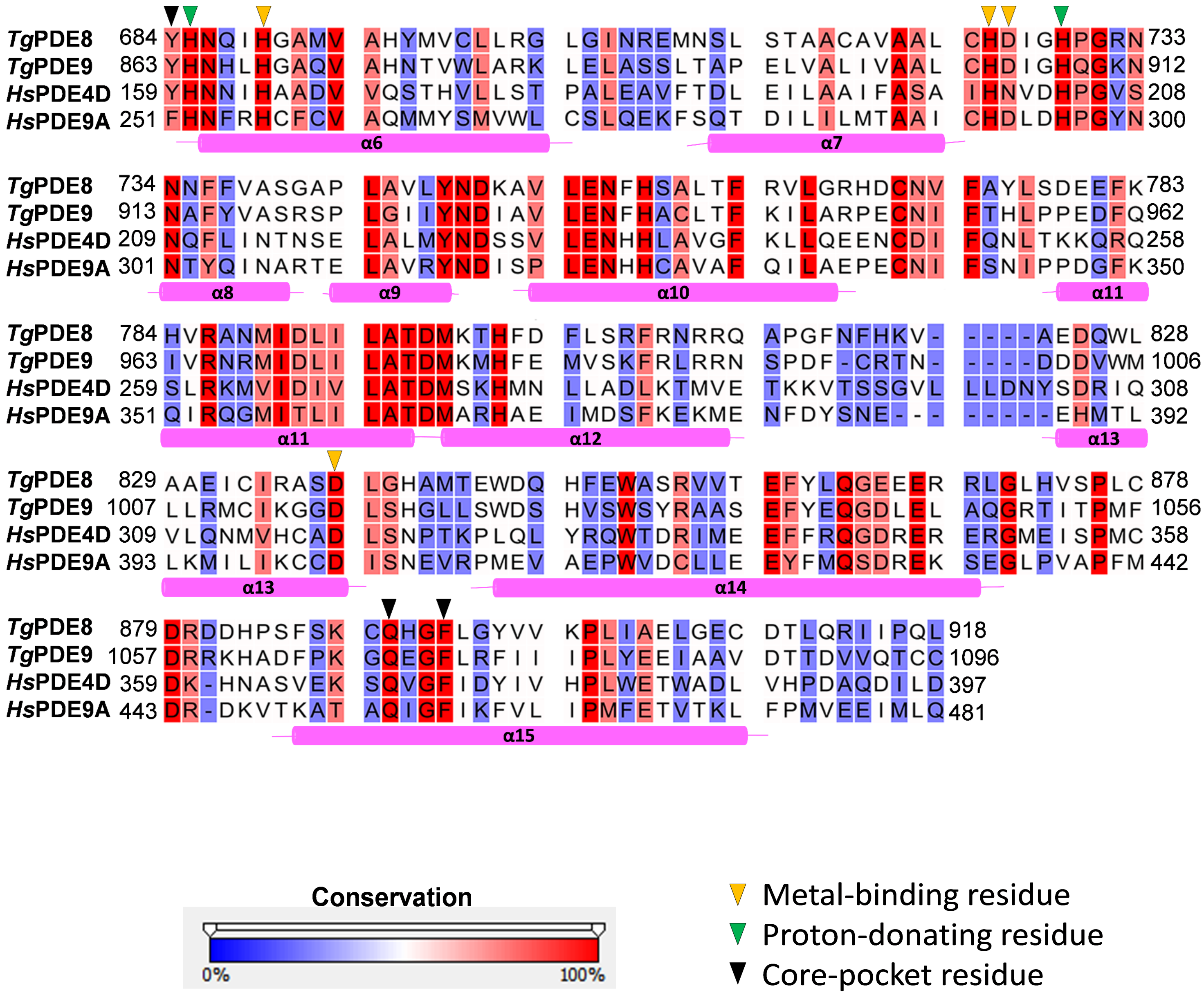

TgPDE8
TgPDE9
HsPDE4D
HsPDE9A
Conservation
0%
100%
Metal-binding residue
Proton-donating residue
Core-pocket residue
